# HFIP-Promoted Synthesis of Substituted Tetrahydrofurans by Reaction of Epoxides with Electron-Rich Alkenes

**DOI:** 10.3390/molecules25153464

**Published:** 2020-07-30

**Authors:** Natalia Llopis, Alejandro Baeza

**Affiliations:** Departamento de Química Orgánica and Instituto de Síntesis Orgánica (ISO), Facultad de Ciencias, Universidad de Alicante. Apdo. 99, E-03080 Alicante, Spain; natalia.llopis@ua.es

**Keywords:** tetrahydrofuranes, fluorinated alcohols, green chemistry

## Abstract

In the present work, the employment of fluorinated alcohols, specifically 1,1,1,3,3,3-hexafluoroisopropanol (HFIP), as solvent and promoter of the catalyst-free synthesis of substituted tetrahydrofuranes through the addition of electron-rich alkenes to epoxydes is described. The unique properties of this fluorinated alcohol, which is very different from their non-fluorinated analogs, allows carrying out this new straightforward protocol under smooth reaction conditions affording the corresponding adducts in moderate yields in the majority of cases. Remarkably, this methodology has allowed the synthesis of new tetrahydrofuran-based spiro compounds as well as tetrahydrofurobenzofuran derivatives. The scope and limitations of the process are also discussed. Mechanistic studies were also performed pointing towards a purely ionic or a S_N_2-type process depending on the nucleophilicity of the alkene employed.

## 1. Introduction

**Figure 1:**
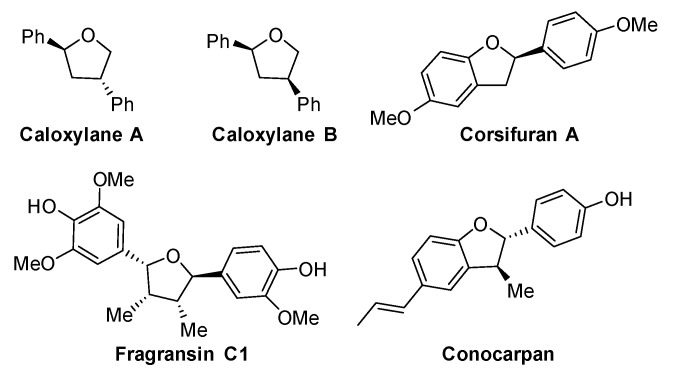
Natural products and bioactive molecules containing tetrahydrofuran moiety.

The substituted tetrahydrofuran structure is present in a wide variety of bioactive natural compounds and has gained considerable interest in pharmaceutical research. In general, natural compounds containing tetrahydrofuran ring derivatives have been found in different classes of terrestrial and marine organisms [1,2,3]. One of these representative examples are Caloxylanes A and B, both isolated from the Caribbean marine sponge *Calyx podatypa* [4,5], or Corsifuran A, isolated from the heartwood of the tree *Thespesia populnea* [6]. Other examples are the lignans Fragransin C1 (among other compounds from the Fragransin family) [1,7] and Conocarpan [1,8], both having demonstrated to exhibit biological activity (Figure 1).

**Scheme 1:**
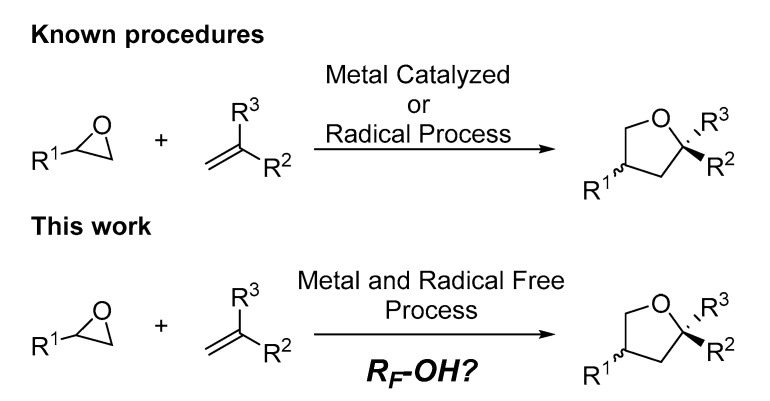
Synthesis of substituted tetrahydrofurans by reaction between alkenes and epoxides.

It is not surprising then that a considerable amount of strategies have been already described in order to gain access to these interesting molecules. Among them, probably the most straightforward method is based on the reaction between alkenes and epoxides, which are commercially available and highly abundant in bulk. This perfect atom-economy route provides direct access to different substituted tetrahydrofurans allowing a wide range of substitution patterns on the structure. However, to the best of our knowledge, only a limited number of publications following this protocol have been reported, being those mainly radical [9] or metal-catalyzed processes (Scheme 1) [10,11,12,13].

In the last years, our research group has become interested in the use of fluorinated alcohols as solvents and promoters of organic reactions [14,15]. The unique chemical and physical properties that fluoroalkyl alcohols have in comparison with their non-fluorinated analogues, such as their high hydrogen bond donor ability, high polarity and ionizing power, and low nucleophilicity values together with the slightly acidic character, make them perfect candidates as promoters of reactions involving ionic processes [16,17,18,19,20,21,22]. On the other hand, fluorinated alcohols have already proven to be efficient promoters in the ring-opening reaction of epoxides with different nucleophiles [23,24,25,26].

With all these precedents in mind, we envisioned a new strategy based on the use of fluorinated alcohols as solvents and reaction promoters in a metal and radical-free ring-opening reaction of epoxides with different electron-rich alkenes as nucleophiles in order to obtain the corresponding substituted tetrahydrofurans in an efficient, cost-effective, and environmentally friendly chemical manner. The results of this investigation are herein described.

## 2. Results and Discussion

Firstly, the reaction between styrene oxide (**1a**) and *α*-methylstyrene (**1b**) was selected as a model in order to obtain the optimal reaction conditions. Different solvents were selected to evaluate their performance as promoters at 45 °C (Table 1, entries 1–4). When water and 2-propanol, which possesses quite high polarity and hydrogen bond ability, were used, the reaction produced the diol **4** as major product and failed (Table 1, entries 1 and 2). Next, 1,1,1,3,3,3-hexafluoroisopropanol (HFIP) and 2,2,2-trifluoroethanol (TFE), as readily available and inexpensive fluorinated alcohols, were tested. As observed in the table, whereas the reaction with HFIP afforded the desired product in high conversion, TFE barely produced tetrahydrofuran **3aa** (Table 1, entries 3 and 4, respectively). This sharp contrast in the performance of both fluorinated alcohols in this transformation can be explained by their different properties. Thus, HFIP has higher acidity (p*K*_a_(TFE) = 12.37, p*K*_a_(HFIP) = 9.30); higher hydrogen bond ability (α_TFE_ = 1.51, α_HFIP_ = 1.96), which can facilitate the activation of epoxide ring; and much lower nucleophilicity (*N*_TFE_ = −2.78, *N*_HFIP_ = −4.23) [16,17,18,19,20,21,22]. This last parameter would explain the obtention of fluoroalkyl ether **5** as major product when TFE was essayed. On the contrary, the corresponding fluorinated ether **6**, derived from HFIP (along with phenylacetaldehyde and acetophenone) was obtained only as by-product. The absence of any solvent was also checked and, as was expected, the reaction did not take place (Table 1, entries 5). Then, efforts to improve the conversion of **3aa** by using a series of HFIP/CH_2_Cl_2_ [16,17,18,19,20,21,22] mixtures were implemented (Table 1, entries 6–8), but turned out to be unsuccessful in all the cases. Lowering the reaction temperature to 25 °C also resulted in a drop in the conversion towards the desired product (Table 1, entry 9). Other changes in reaction stoichiometry were also essayed but did not produce any amelioration. After the search for the best conditions, those described in entry 3, involving the use of HFIP at 45 °C, were selected as optimal, realizing that reaction was complete in less than 10 h.

**Scheme 2:**
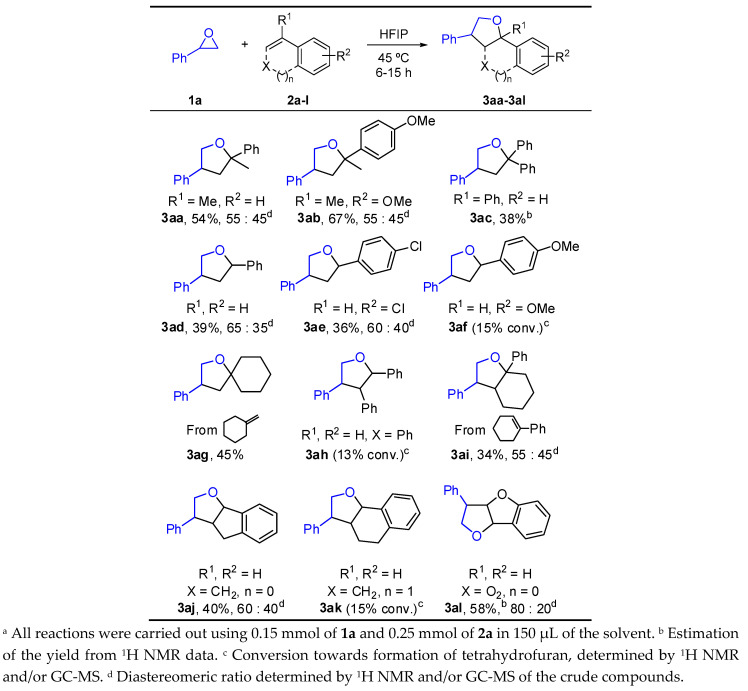
Reaction between styrene oxide and electron-rich alkenes ^a^.

With these conditions in hand, the scope of the reaction was next investigated. Different electron-rich alkenes were employed as nucleophiles for the ring opening reaction of styrene oxide (Scheme 2). It is important to mention that in the majority of cases, the ring-opening reaction was highly regioselective, but when a mixture of diastereoisomers was obtained, low diastereoselective ratios were observed. First, a selection of substituted styrenes was chosen. As mentioned above, *α*-methylstyrene (**2a**) produced the corresponding tetrahydrofuran **3aa** in moderate isolated yield. Better results were observed when a more electron-rich alkene, **2b**, was employed; reaching up to 67% yield for **3ab**. The more sterically crowded 1,1-diphenylethylene (**2c**) gave the corresponding product in only modest yield. In this case, some amount (25%) of the other regioisomer was also obtained, probably due to the mentioned steric hindrance. Next, styrene was essayed obtaining the corresponding caloxylane **3ad** (as 65:35 mixture of diastereosiomers) in 39% yield. Similar results were obtained when 4-chlorostyrene was employed. Surprisingly, the more electron-rich alkene, 4-methoxystyrene (**2f**), gave rise to the corresponding product in low yields. At this point, it is worth mentioning that in the majority of the styrenes employed the presence of dimers or trimers of the styrene were detected by GC-MS, thus lowering the yield of the process. Methylenecyclohexane (**2g**) was next tested, obtaining spiro-compound **3ag** in modest yield. Stilbenes were also submitted to the reaction conditions but failed and only a low conversion was observed when the *cis*-isomer was employed. Trisubstituted alkenes such as 1-phenylcyclohexene (**2i**) were also taken into account, obtaining the interesting octahydrobenzofuran derivative **3ai** in 34% yield. Finally, benzocondensed alkenes were also essayed. Thus, whereas indene produced the corresponding product **3aj** in modest yield, the reaction with 1,2-dihydronaphthalene barely worked. Finally, when benzofuran (**2l**) was employed as alkene, the corresponding tetrahydrofuro[3,2-*b*]benzofuran derivative **3al**, arising from the attack of benzofuran through its 2-position onto the epoxide, obtained with moderate yield and quite good diastereoselectivity. It is important to remark that in all the cases ether **6** along with the products coming from the Meinwald rearrangement [27] of the epoxide (benzaldehyde and acetophenone, the first one with higher proportion) were obtained as by-products.

In order to further expand the scope of the reaction, other epoxides were tested with those alkenes that provided the best results. First, *α*-methylstyrene oxide (**1b**) was evaluated. Good yields were achieved when **2a** and **2b** were the alkenes employed. However, modest yields were only achieved when ethylenecyclohexane (**2g**) and benzofuran (**2l**) were used. Next, when 1-phenylcyclohexene oxide (**1c**) was the substrate submitted to the reaction with the same alkenes, modest yields were obtained for adducts **3ca** and **3cb**. Unfortunately, the reaction with **2g** did not work. Although, in a modest 38% yield, benzofuran (**2l**) rendered the interesting tetracyclic compound **3cl**. Finally, commercial available ethyl 3-methyl-3-phenylglycidate (**1d**) was also tested. It is remarkable that, contrary to the normal trend observed concerning the low diastereoselectivity achieved in previous cases, moderate to good diastereoselectivities were achieved when **1d** was the substrate employed. Modest yield was achieved when *α*-methylstyrene (**2a**) was employed, rendering the densely substituted **3da** in 43% yield. However, the more electron-rich alkene **2b** did not produce satisfactory results, being the dimerization and trimerization product of the alkene the major products observed by GC-MS. To our surprise, alkene **2c** gave rise to the corresponding tetrahydrofuran **3dc** in 60% yield and a 90:10 diastereomeric ratio. Encouraged by this result, styrene was also employed obtaining **3dd** in modest yield. Unfortunately, the reaction with alkenes **2g** and **2j** turned out to be unsuccessful and low conversions towards the desired products were obtained. Finally, benzofuran (**2l**) rendered the corresponding tricyclic compound in a modest 44% yield. It is worth mentioning that other epoxides such as cyclohexene oxide, 1-octene oxide, indene oxide, and *cis*- and *trans*-stilbene oxide were also submitted to the reaction with the alkenes depicted in Table 2; however, to our regret the reaction failed.

**Scheme 3:**
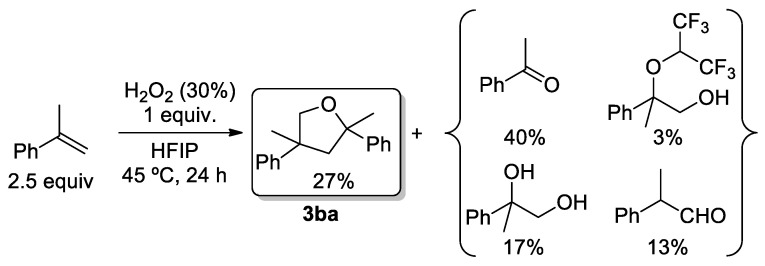
One-pot oxidation/ring opening reaction.

At this point, as epoxides are synthesized from alkenes, and fluorinated alcohols have proven to be efficient mediators in the oxidation of alkenes using H_2_O_2_ [28], we decided to explore the possibility of performing the HFIP-promoted alkene oxidation/ring opening of epoxides in a one-pot reaction (Scheme 3). For such purpose, an excess of *α*-methylstyrene (**2a**) was treated with 1 equivalent of H_2_O_2_ (30%) for 24 h. After this time among a myriad of products detected by GC-MS coming from the ring opening of the epoxide with H_2_O or HFIP, 2-phenylpropanaldehyde from Meinwald rearrangement and acetophenone from oxidative cleavage of the alkene, tetrahydrofuran **3aa** was observed in 27% conv. Although the product was obtained in low amount, it can be seen as a proof of concept that substituted tetrahydrofurans can be easily obtained from readily available materials as styrenes.

**Scheme 4:**
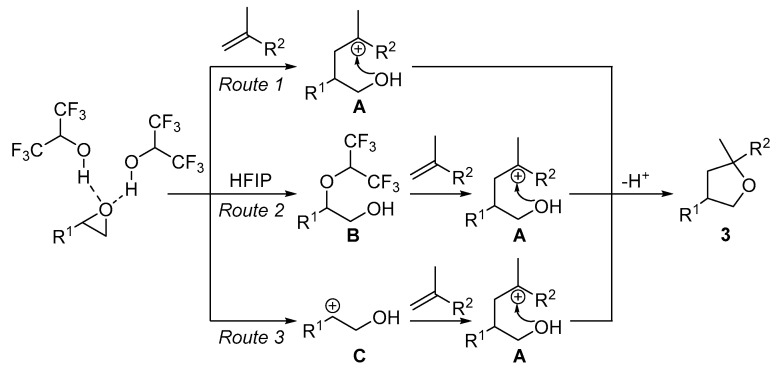
Possible operating reaction mechanisms.

**Scheme 5:**
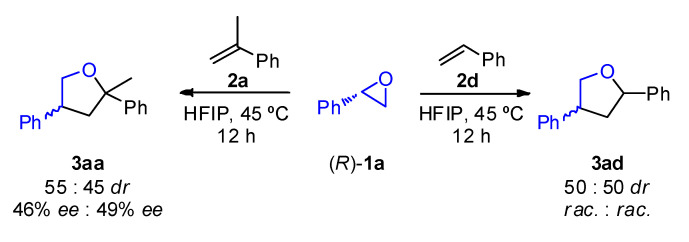
Mechanism elucidation tests.

Concerning the reaction mechanism, three possible scenarios were taken into account (Scheme 4). In route 1, direct nucleophilic attack of the alkene onto activated epoxide would occur rendering intermediate A, which cyclizes to afford the corresponding tetrahydrofuran. In route 2, intermediate A is obtained as consequence of a double nucleophilic attack, the first one carried out by the more abundant HFIP followed by a nucleophilic substitution. In the route 3, carbocationic intermediate C is formed, which could be stabilized by the formation of ionic pair or by other electrostatic interactions with HFIP. Route 2 was soon discarded due to the fact that intermediate B has been observed in the reaction and if this would have been the operating route, longer reaction times would render higher conversions, which did not happen. Nevertheless, ether **6** was synthesized by reacting **1a** with HFIP by 8 h at room temperature, and after a quick purification, was allowed to react with *α*-methylstyrene (**2a**) for 24 h under the optimized conditions. After this time, no reaction was observed. In order to find out whether route 1 or 3 was operating in the process, we decided to carry out the reaction using enantiopure (*R*)-styrene oxide and *α*-methylstyrene (**2a**) and styrene (**2d**) as alkenes (Scheme 5). Thus, if route 1 is the one taking place, the configuration of the stereocenter will be somehow preserved. As depicted in Scheme 4, corroborated by chiral HPLC analysis (see Supplementary Materials for further details), when styrene (**2d**) was the nucleophile, the stereochemistry of the chiral center was lost giving a racemic mixture in each diastereoisomer of caloxylane (**3ad**). However, the better nucleophile *α*-methylstyrene (**2a**) gave rise to a mixture of diastereoisomers both presenting a loss of enantiopurity in the chiral centre (46% *ee* and 49% *ee*, respectively), which was determined by chiral HPLC analysis (see Supplementary Materials for further details). Therefore, these experimental evidences point that the mechanism of the reaction seemingly is highly dependent on the nucleophilicity of the alkene employed. Thus, whereas styrene (**2d**) apparently follows a purely ionic route (S_N_1-type mechanism (route 3, Scheme 4)) in the *α*-methylstyrene (**2a**) case, predominantly a S_N_2-type pathway (route 1, Scheme 3) is operating.

## 3. Materials and Methods

All reagents and solvents were obtained commercially and used without further purification. Substrates that were not commercially available were synthesized according to known literature procedures. NMR spectra were performed on a Bruker AV-300 or Bruker AV-400 (Bruker Corporation, Billerica, MA, USA) using CDCl_3_ as solvent and TMS as internal standard unless otherwise stated. Conversions and low-resolution mass spectra (MS) of the tetrahydrofurans 3 were recorded in the electron impact mode (EI, 70 eV, He as carrier phase) using an Agilent GC/MS 5973 Network Mass Selective Detector spectrometer apparatus equipped with a HP-5MS column (30 m × 0.25 mm) (Agilent technologies, Bilbao, Spain) and giving fragment ions in *m*/*z* with relative intensities (%) in parentheses. High-resolution mass spectra (HRMS) were obtained on an Agilent 7200 Quadrupole-Time of Flight apparatus (Q-TOF) (Agilent Technologies), with the ionization employed being electron impact (EI). Chiral HPLC analysis was performed in an Agilent 1100 Series HPLC equipped with a G1315B diode array detector and a Quat Pump G1311A (Agilent Technologies) equipped with the corresponding Daicel chiral column. Analytical TLC was performed on Merck silica gel plates and the spots visualized with UV light at 254 nm (Merck millipore, Darmstadt, Germany). Flash chromatography employed Merck silica gel 60 (0.040–0.063 mm). Silica gel 60 F_254_ containing gypsum was employed for preparative layer chromatography (Merck millipore).

### General Procedure for the HFIP-Promoted Synthesis of Substituted Tetrahydrofurans

In a capped tube, onto a mixture of the corresponding epoxide (0.15 mmol) and alkene (0.25 mmol), HFIP (150 µL) was added in one portion. The reaction was then stirred at 45 °C for 6–15 h, until the reaction was judged to be completed (no starting epoxide remaining) by GC-MS. After this time, solvent was evaporated and the crude material was directly purified by flash chromatography or preparative TLC.

2-Methyl-2,4-diphenyltetrahydrofuran (**3aa**) [11]: yellow oil; purification by flash chromatography (hexane/EtOAc), 54% yield; (*cis/trans*) = 55:45; ^1^H NMR (300 MHz, CDCl_3_): *cis* isomer: δ_H_ = 7.52–7.45 (m, 4H), 7.43–7.28 (m, 10H), 7.27–7.16 (m, 6H), 4.42 (t, *J* = 7.6 Hz, 1H), 3.84 (dd, *J* = 10.0, 8.2 Hz, 1H), 3.70 (tt, *J* = 10.3, 7.7 Hz, 1H), 2.41–2.15 (m, 2H), 1.63 (s, 3H) ppm; further signals for the *trans* isomer: δ_H_ = 4.35 (t, *J* = 8.4 Hz, 1H), 4.00 (t, *J* = 8.7 Hz, 1H), 3.31 (ddd, *J* = 15.9, 11.3, 8.6 Hz, 1H), 2.81–2.60 (m, 2H), 1.68 (s, 3H) ppm; ^13^C NMR (101 MHz, CDCl_3_): *cis* isomer: δ_C_ = 148.9, 140.8, 128.5, 128.3, 127.4, 126.6, 126.5, 124.5, 74.4, 47.9, 45.8, 30.6, ppm; further signals for *trans* isomer: 147.6, 141.6, 128.5, 128.2, 127.3, 126.6, 126.4, 124.7, 73.9, 48.3, 44.6, 30.2 ppm; MS (EI): *m*/*z* 238 (M^+^, 0.31%), 224 (19), 223 (100), 193 (12), 117 (27), 115 (18), 105 (90), 91 (16), 77 (18). Chiral HPLC analysis: Chiralpak IA column, Hexane/iPrOH 99:1, flow rate = 0.2 mL/min, λ = 210 nm, retention times: = 25.5 and 26.5 min. (major diastereosiomer) and 27.4 and 28.4 min. (minor diastereoisomer).

2-(4-Methoxyphenyl)-2-methyl-4-phenyl tetrahydrofuran (**3ab**): orange oil; purification by flash chromatography (hexane/EtOAc), 67% yield; (*cis*/*trans*) = 55:45; ^1^H NMR (300 MHz, CDCl_3_): *cis* isomer: δ_H_ = 7.43–7.37 (m, 3H), 7.33–7.28 (m, 3H), 7.27–7.16 (m, 6H), 6.95–6.93 (m, 2H), 6.92–6.90 (m, 2H), 4.40 (t, *J* = 7.9 Hz, 1H), 3.84 (s, 3H), 3.82 (dd, *J* = 3.5, 1.9 Hz, 1H), 3.76–3.64 (m, 1H), 2.63 (dd, *J* = 12.4, 8.0 Hz, 1H), 2.32 (dd, *J* = 12.3, 10.5 Hz, 1H), 2.24–2.17 (m, 1H), 1.61 (s, 3H) ppm; further signals for the *trans* isomer: δ_H_ = 4.33 (t, *J* = 8.4 Hz, 1H), 3.98 (t, *J* = 8.6 Hz, 1H), 3.85 (s, 3H), 3.39–3.25 (m, 1H), 2.71 (dd, *J* = 12.1, 7.1 Hz, 1H), 2.32 (dd, *J* = 12.3, 10.5 Hz, 1H), 1.66 (s, 3H) ppm; ^13^C NMR (101 MHz, CDCl_3_): mixture of isomers, δ_C_ = 158.3, 158.2, 141.7, 141.1, 141.0, 139.7, 128.5, 128.5, 127.4, 127.3, 126.6, 126.6, 125.8, 125.7, 113.6, 113.5, 85.2, 84.7, 74.4, 73.9, 55.3, 55.2, 48.3, 48.1, 45.8, 44.6, 30.6, 30.3 ppm; MS (EI): *m*/*z* 268 (M^+^, 6%), 254 (17), 253 (93), 135 (100), 117 (14), 91 (11); HRMS (GC/MS-EI/Q-TOF): *m*/*z* calcd. for C_18_H_20_O_2_ 268.1463, found 268.1463.

2,2,4-Triphenyltetrahydrofuran (**3ac**): yellow oil; purification by flash chromatography (hexane/EtOAc), 38% estimated yield (not purely isolated); ^1^H NMR (300 MHz, CDCl_3_): δ_H_ = 7.54–7.49 (m, 4H), 7.37–7.34 (m, 4H), 7.34–7.30 (m, 4H), 7.27–7.24 (m, *J* = 1.6 Hz, 2H), 7.24–7.22 (m, 1H), 4.48 (t, *J* = 8.4 Hz, 1H), 4.07 (t, *J* = 8.7 Hz, 1H), 3.61–3.43 (m, *J* = 16.0, 11.0, 5.2 Hz, 1H), 3.23 (dd, *J* = 12.3, 7.1 Hz, 1H) ppm; MS (EI): *m*/*z* 300 (M^+^, 55%), 270 (15), 224 (71), 223 (100), 192 (21), 191 (13), 179 (13), 178 (15), 165 (21), 118 (34), 117 (42), 115 (12), 105 (96), 91 (14), 77 (27); HRMS (GC/MS-EI/Q-TOF): *m*/*z* calcd. for C_22_H_20_O 300.1514, found 300.1509.

2,4-Diphenyltetrahydrofuran (Caloxylane A and B) (**3ad**) [11]: yellow oil; purification by flash chromatography (hexane/EtOAc), 39% yield; (*cis/trans*) = 65:35; ^1^H NMR (300 MHz, CDCl_3_): *cis* isomer: δ_H_ = 7.49–7.29 (m, 20H), 5.11 (dd, *J* = 10.2, 5.7 Hz, 1H), 4.39 (t, *J* = 8.2 Hz, 1H), 4.05 (t, *J* = 8.5 Hz, 1H), 3.74–3.63 (m, 1H), 2.85–2.72 (m, 1H), 2.05 (q, *J* = 10.5, 1.9 Hz, 1H) ppm; further signals for the *trans* isomer: δ_H_ = 5.26 (dd, *J* = 7.7, 5.8 Hz, 1H), 4.50 (t, *J* = 8.5, 7.4 Hz, 1H), 3.98 (t, *J* = 8.2 Hz, 1H), 3.63–3.51 (m, 1H), 2.57–2.45 (m, 1H), 2.36 (q, *J* = 12.5, 8.3, 5.8 Hz, 1H) ppm; ^13^C NMR (75 MHz, CDCl_3_): *cis* isomer: δ_C_ = 142.6, 141.7, 128.6, 128.4, 127.4, 127.2, 125.7, 81.8, 75.1, 46.0, 43.7, pm; further signals for the *trans* isomer: δ_C_ = 143.6, 142.0, 128.6, 128.3, 127.3, 127.1, 125.5, 80.6, 75.1, 44.4, 42.7 ppm; MS (EI): *m*/*z* 224 (M^+^, 34%), 195 (14), 194 (93), 193 (100), 179 (58), 178 (89), 165 (13), 146 (27), 133 (34), 120 (27), 117 (90), 115 (57), 105 (45), 91 (48), 77 (30). Chiral HPLC analysis: Chiralcel OD-H column, Hexane/iPrOH 99:1, flow rate = 0.7 mL/min, λ = 210 nm, retention times: = 23.1 and 23.2 min. (major diastereosiomer) and 28.0 and 35.4 min. (minor diastereoisomer).

2-(4-Chlorophenyl)-4-phenyltetrahydrofuran (**3ae**) [11]: yellow oil; purification by flash chromatography (hexane/EtOAc), 36% yield; (*cis*/*trans*) = 60:40; ^1^H NMR (400 MHz, CDCl_3_): *cis* isomer: δ_H_ = 7.40–7.30 (m, 9H), 5.07 (dd, *J* = 10.1, 5.8 Hz, 1H), 4.38 (t, *J* = 8.2 Hz, 1H), 4.03 (t, *J* = 8.5 Hz, 1H), 3.78–3.61 (m, 1H), 2.86–2.72 (m, 1H), 1.98 (dd, *J* = 12.4, 10.4 Hz, 1H) ppm; further signals for the *trans* isomer: δ_H_ = 5.22 (dd, *J* = 7.7, 5.9 Hz, 1H), 4.47 (dd, *J* = 8.4, 7.5 Hz, 1H), 3.97 (t, *J* = 8.2 Hz, 1H), 3.54 (t, *J* = 7.6 Hz, 1H), 2.50 (dt, *J* = 12.6, 7.7 Hz, 1H), 2.35–2.24 (m, 1H) ppm; ^13^C NMR (101 MHz, CDCl_3_): *cis* isomer: δ_C_ = 141.5, 141.2, 133.0, 129.9, 128.8, 128.6, 128.3, 128.2, 127.3, 127.1, 126.7, 81.1, 75.1, 45.9, 43.8 ppm; further signals for the *trans* isomer: δ_C_ = 142.1, 142.7, 132.8, 129.8, 128.7, 128.5, 128.3, 128.1, 127.2, 126.9, 126.7, 126.4, 79.9, 72.7, 44.3, 42.7 ppm; MS (EI): *m*/*z* 258 (M^+^, 18%), 228 (25), 193 (100), 180 (15), 178 (22), 167 (22), 154 (16), 139 (27), 117 (64), 115 (54), 104 (17), 91 (27), 77 (13).

3-Phenyl-1-oxaspiro[4.5]decane (**3ag**): yellow solid, purification by flash chromatography (hexane/EtOAc), 45% yield; ^1^H NMR (400 MHz, CDCl_3_): δ_H_ = 7.36–7.31 (m, 3H), 7.27–7.23 (m, 2H), 4.23 (t, *J* = 8.0 Hz, 1H), 3.80 (t, *J* = 17.6 Hz, 1H), 3.51 (tt, *J* = 17.6, 8.8 Hz, 1H), 2.30 (dd, *J* = 12.4, 8.2 Hz, 1H), 1.78 (dd, *J* = 12.4, 10.5 Hz, 1H), 1.74–1.48 (m, 10H) ppm; ^13^C NMR (126 MHz, CDCl_3_): δ_C_ = 141.9, 128.6, 128.5, 128.1, 127.3, 126.5, 83.3, 72.9, 45.0, 38.3, 37.3, 25.61, 23.8, 23.8 ppm; MS (EI): *m*/*z* 216 (M^+^, 55%), 174 (25), 173 (100), 160 (40), 118 (18), 117 (28), 104 (41), 91 (26), 55 (73); HRMS (GC/MS-EI/Q-TOF): *m*/*z* calcd. for C_15_H_20_O 216.1514, found 216.1514.

3,7a-Diphenyloctahydrobenzofuran (**3ai**): orange oil; purification by flash chromatography (hexane/EtOAc); 34% yield; diastereomeric ratio = 55:45; ^1^H NMR (300 MHz, CDCl_3_): *major* isomer: δ_H_ = 7.58 (dd, *J* = 8.5, 1.1 Hz, 2H), 7.54–7.48 (m, 2H), 7.44–7.35 (m, 6H), 7.34–7.28 (m, 4H), 7.23 (m, 6H), 7.16–7.08 (m, 2H), 4.40 (m, 2H), 4.00–3.91 (m, 1H), 3.59 (m, 1H), 2.67 (dd, *J* = 12.0, 5.5 Hz, 1H), 2.13–1.85 (m, 4H), 1.83–1.52 (m, 12H) ppm; furher signals for the *minor* isomer: δ_H_ = 4.27 (t, *J* = 8.6 Hz, 1H), 3.42 (td, *J* = 9.6, 5.5 Hz, 1H), 2.57 (dt, *J* = 11.4, 5.7 Hz, 1H) ppm; ^13^C NMR (75 MHz, CDCl_3_): *major* isomer: δ_C_ = 148.6, 141.2, 128.6, 128.2, 128.1, 126.9, 126.4, 125.9, 84.8, 72.6, 51.2, 46.6, 35.8, 24.2, 21.9, 20.0 ppm; further signals for *minor* isomer: δ_C_ = 146.5, 138.2, 128.3, 128.1, 127.9, 126.7, 126.2, 124.5, 86.8, 67.3, 49.4, 46.4, 38.3, 24.6, 22.1, 20.0 ppm; MS (EI): *m*/*z* 278 (M^+^, 85%), 236 (19), 235 (100), 221 (18), 115 (14), 105 (67), 91 (25), 77 (18); HRMS (GC/MS-EI/Q-TOF): *m*/*z* calcd. for C_20_H_22_O 278.1671, found 278.1667.

3-Phenyl-3, 3a, 4, 8b-tetrahydro-2H-indeno [1,2-b]furan (**3aj**) [11]: yellow oil; purification by flash chromatography (hexane/EtOAc), 40% yield; (*cis*/*trans*) = 60:40; ^1^H NMR (300 MHz, CDCl_3_): *cis* isomer: δ_H_ = 7.53–7.47 (m, 2H), 7.39–7.29 (m, 8H), 5.73 (d, *J* = 7.2 Hz, 1H), 4.13 (dd, *J* = 8.6, 6.7 Hz, 1H), 3.92 (dd, *J* = 8.6, 7.7 Hz, 1H), 3.26–3.16 (m, 2H), 3.12–3.03 (m, 1H), 3.01–2.91 (m, 1H) ppm; further signals for the *trans* isomer: δ_H_ = 7.51–7.46 (m, 1H), 7.36–7.24 (m, 6H), 7.14–7.09 (m, 2H), 5.69 (d, *J* = 6.8 Hz, 1H), 4.27–4.21 (m, 1H), 3.82–3.73 (m, 2H), 3.59–3.47 (m, 1H), 2.83 (dd, *J* = 17.4, 9.3 Hz, 1H), 2.60 (dd, *J* = 17.4, 4.8 Hz, 1H) ppm; ^13^C NMR (75 MHz, CDCl_3_): mixture of isomers, δ_C_ = 142.5, 141.9, 141.7, 141.6, 128.8, 128.7, 128.4, 128.4, 127.5, 127.2, 127.0, 126.7, 126.5, 125.6, 125.2, 124.4, 87.9, 87.8, 74.6, 68.2, 53.3, 50.1, 48.5, 45.5, 38.7, 36.6 ppm; MS (EI): *m*/*z* 236 (M^+^, 34%), 207 (17), 206 (100), 205 (27), 128 (20), 115 (27), 91 (86).

3-Phenyl-2,3,3a,8b-tetrahydrofuro[3,2-b]benzofuran (**3al**): yellow oil; purification by flash chromatography (hexane/EtOAc), 40% estimated yield, mixture of isomers (not purely isolated); ^1^H NMR (400 MHz, CDCl_3_): δ_H_ = 7.50–7.46 (m, 1H), 7.40–7.38 (m, 3H), 7.35–7.30 (m, 3H), 6.99 (td, *J* = 7.4, 0.9 Hz, 1H), 6.88 (d, *J* = 8.3 Hz, 1H), 5.81 (d, *J* = 6.0 Hz, 1H), 5.19 (dd, *J* = 6.0, 1.2 Hz, 1H), 4.08 (dd, *J* = 9.1, 2.3 Hz, 1H), 3.96 (dd, *J* = 9.1, 5.5 Hz, 1H), 3.67 (d, *J* = 5.5 Hz, 1H) ppm; MS (EI): *m*/*z* 238 (M^+^, 34%), 220 (66), 219 (43), 208 (45), 207 (100), 191 (15), 189 (17), 178 (19), 165 (13), 131 (24), 117 (12); HRMS (GC/MS-EI/Q-TOF): *m*/*z* calcd. for C_16_H_14_O 238.0994, found 238.0990.

2,4–Dimethyl–2,4–diphenyltetrahydrofuran (**3ba**) [29]: yellow solid; purification by flash chromatography (hexane/EtOAc), 62% yield; (*cis/trans*) = 50:50; the *cis* isomer is highlighted in bold; ^1^H NMR (300 MHz, CDCl_3_): δ_H_ = 7.54–7.49 (m, 2H), 7.45–7.39 (m, 2H), 7.39–7.30 (m, 8H), 7.26–7.21 (m, 4H), 7.20–7.10 (m, 4H), 4.30 (d, *J* = 8.6 Hz, 1H), 4.10 (dd, *J* = 8.4, 1.0 Hz, 1H), 4.06 (d, *J* = 8.5 Hz, 1H), 3.96 (dd, *J* = 8.4, 0.6 Hz, 1H), 2.72 (d, *J* = 12.6 Hz, 1H), 2.58 (s, 2H), 2.47 (dd, *J* = 12.6, 1.1 Hz, 1H), 1.70 (s, 3H), 1.59 (s, 6H), 1.50 (s, 3H) ppm; MS (EI): *m*/*z* 252(M^+^, 0.08%), 237 (100), 207 (12), 129 (13), 117 (29), 105 (97), 91 (14), 77 (15).

2–(4–Methoxyphenyl)–2,4–dimethyl-4-phenyl tetrahydrofuran (**3bb**): white solid; purification by flash chromatography (hexane/EtOAc), 58% yield; (*cis/trans*) = 45:55; the cis isomer is highlighted in bold; ^1^H NMR (300 MHz, CDCl_3_): δ_H_ = 7.36–7.30 (m, 3H), 7.27–7.22 (m, 2H), 7.16–7.09 (m, 2H), 6.87–6.82 (m, 2H), 4.28 (d, *J* = 8.6 Hz, 1H), 4.07 (d, *J* = 9.1 Hz, 1H), 4.03 (s, 1H), 3.95 (d, *J* = 8.4 Hz, 1H), 3.84 (s, 3H), 3.80 (s, 3H), 2.70 (d, *J* = 12.6 Hz, 1H), 2.55 (d, *J* = 2.2 Hz, 1H), 2.42 (d, *J* = 12.5 Hz, 1H), 1.67 (s, 3H), 1.58 (s, 3H), 1.48 (s, 3H), 1.23 (s, 3H) ppm; Only major isomer is given, ^13^C NMR (101 MHz, CDCl_3_): δ_H_ = 157.9, 147.7, 141.6, 128.3, 126.0, 125.9, 125.6, 113.5, 84.6, 77.8, 55.3, 53.9, 48.9, 32.8, 29.9 ppm; MS (EI): *m*/*z* 282(M^+^, 9%), 268 (16), 267 (83), 135 (100), 117 (13); HRMS (GC/MS-EI/Q-TOF): *m*/*z* calcd. for C_19_H_22_O_2_ 282.1620, found 282.1620.

3-Methyl-3-phenyl-1-oxaspiro[4,5]decane (**3bg**): colourless oil; purification by flash chromatography (hexane/EtOAc), 30% estimated yield (not purely isolated); ^1^H NMR (300 MHz, CDCl_3_): δ_H_ = 7.36–7.30 (m, 4H), 7.26–7.23 (m, 1H), 4.05 (d, *J* = 8.7 Hz, 1H), 3.94 (d, *J* = 8.5 Hz, 1H), 2.12 (d, *J* = 12.6 Hz, 1H), 1.98 (dd, *J* = 12.6, 0.7 Hz, 1H), 1.79–1.7 (m, 5H), 1.61 (d, *J* = 2.7 Hz, 2H) 1.51 (d, *J* = 6.0 Hz, 3H), 1.46 (s, 3H) ppm; MS (EI): *m*/*z* 230(M^+^, 43%), 216 (16), 215 (100), 187 (59), 118 (19), 117 (25), 91 (14), 55 (30); HRMS (GC/MS-EI/Q-TOF): *m*/*z* calcd. for C_16_H_22_O 230.1671, found 230.1672.

3-Methyl-3-phenyl-2,3,3a,8b-tetrahydrofuro [3,2-b] benzofuran (**3bl**): Inseparable mixture of regioisomers. The major isomer data is highlighted in bold. Yellow solid; purification by flash chromatography (hexane/EtOAc), 35% estimated yield (not purely isolated); ^1^H NMR (400 MHz, CDCl_3_): δ_H_ = 7.60–7.49 (m, 3H), 7.48–7.38 (m, 3H), 7.38–7.30 (m, 8H), 7.27–7.21 (m, 2H), 7.00–6.88 (m,2H), 5.60 (d, *J* = 6.0 Hz, 1H), 5.15 (d, *J* = 6.0 Hz, 1H), 4.31 (d, *J* = 9.3 Hz, 1H), 4.19 (d, *J* = 12.4 Hz, 1H), 3.91 (d, *J* = 5.7 Hz, 1H), 3.82 (d, *J* = 12.2 Hz, 1H), 3.64 (d, *J* = 9.3 Hz, 1H), 3.60 (d, *J* = 12.4 Hz, 1H), 1.61 (s, 3H), 1.52 (s, 3H), ppm; MS (EI): *m*/*z* 252 (M^+^, 71%), 237 (14), 221 (15), 207 (100), 194 (20), 178 (15), 145 (23), 131 (41), 129 (11), 118 (36), 115 (16), 105 (14), 91 (20), 89(15), 77 (14); HRMS (GC/MS-EI/Q-TOF): *m*/*z* calcd. for C_17_H_16_O_2_ 252.1150, found 252.1149.

4a-Phenyl-1,2,3,4,4a,4b,9b,10a-octahydrobenzofuro[3,2-b]benzofuran (**3cl**): white solid; purification by flash chromatography (hexane/EtOAc), 38% yield; diastereomeric ratio = 55:45; ^1^H NMR (400 MHz, CDCl_3_): δ_H_ = 7.53–7.47 (m, 3H), 7.45–7.34 (m, 3H), 7.34–7.30 (m, 1H), 7.02–6.90 (m, 2H), 5.54 (d, *J* = 7.8 Hz, 1H), 5.15 (d, *J* = 7.9 Hz, 1H), 4.43 (t, *J* = 2.5 Hz, 1H), 2.17–2.03 (m, 1H), 2.00–1.85 (m, 1H), 1.51–1.39 (m, 3H), 1.05–0.82(m, 3H) ppm; ^13^C NMR (400 MHz, CDCl_3_): δ_C_ =130.7, 130.4, 128.5, 128.2, 128.0, 127.0, 126.4, 126.3, 126.2, 126.0, 120.9, 120.7, 110.0, 109.7, 96.8, 93.9, 80.7, 80.5, 78.9, 77.2, 75.3, 32.2, 29.7, 28.7, 26.5, 25.1, 21.1, 20.4, 20.2 ppm; MS (EI): *m*/*z* 292 (M^+^, 17%), 274 (20), 208 (17), 207 (100), 194 (25), 91 (11); HRMS (GC/MS-EI/Q-TOF): *m*/*z* calcd. for C_20_H_20_O_2_ 292.1463, found 292.1460.

Ethyl 3,5-dimethyl-3,5-diphenyltetrahydrofuran-2-carboxylate (**3da**): yellow solid; purification by flash chromatography (hexane/EtOAc), 43% yield; diastereomeric ratio = 65:35; ^1^H NMR (300 MHz, CDCl_3_): δ_H_ = 7.68–7.62 (m, 2H), 7.54–7.49 (m, 2H), 7.42–7.34 (m, 6H), 7.26 (d, *J* = 2.0 Hz, 1H), 5.08 (s, 1H), 4.21 (qd, *J* = 7.1, 1.5 Hz, 2H), 2.75 (d, *J* = 12.9 Hz, 1H), 2.65 (d, *J* = 12.8 Hz, 1H), 1.61 (s, 3H), 1.23 (t, *J* = 7.1 Hz, 3H) 1.22 (t, *J* = 7.1 Hz, 3H) ppm; ^13^C NMR (101 MHz, CDCl_3_): only major isomer is given, δ_C_ = 170.6, 148.7, 145.4, 128.3, 128.1, 126.4, 126.3, 126.3, 124.6, 84.7, 84.3, 60.7, 56.6, 31.8, 29.7, 23.5, 14.2 ppm; MS (EI): *m*/*z* 324 (M^+^, 0.13%), 309 (86), 251 (48), 233 (18), 207 (27), 173 (14), 133 (17), 129 (16), 105 (100), 91 (15), 77 (15); HRMS (GC/MS-EI/Q-TOF): *m*/*z* calcd. for C_21_H_24_O_3_ 324.1725, found 324.1715.

Ethyl 3-methyl-3,5,5-triphenyltetrahydrofuran-2-carboxylate (**3dc**): yellow oil; purification by flash chromatography (hexane/EtOAc), 60% yield; diastereomeric ratio = 90:10; ^1^H NMR (300 MHz, CDCl_3_): δ_H_ = 7.70–7.64 (m, 3H), 7.52–7.48 (m, 3H), 7.39–7.30 (m, 6H), 7.24–7.18 (m, 3H), 4.96 (s, 1H), 4.14 (q, *J* = 7.1 Hz, 2H), 3.33 (d, *J* = 13.1 Hz, 1H), 3.01 (d, *J* = 13.0 Hz, 1H), 1.34 (s, 3H), 1.20 (s, 3H) ppm; ^13^C NMR (101 MHz, CDCl_3_): δ_C_ = 170.3, 147.2, 147.0, 145.1, 128.5, 128.3, 128.2, 128.1, 127.8, 126.8, 126.6, 126.5, 126.4, 126.3, 125.7, 125.5, 125.1, 87.6, 85.1, 60.7, 56.0, 51.2, 23.9, 14.2 ppm; MS (EI): *m*/*z* 386 (M^+^, 0.75%), 314 (15), 313 (59), 309 (45), 295 (24), 269 (26), 206 (12), 196 (75), 191 (30), 181 (12), 178 (16), 167 (86), 165 (30), 133 (24), 105 (100), 91 (1), 77 (19); HRMS (GC/MS-EI/Q-TOF): *m*/*z* calcd. for C_26_H_26_O_3_ 386.1882, found 386.1884.

Ethyl-3-methyl-3,5-diphenyltetrahydrofuran-2-carboxylate (**3dd**): orange solid; purification by flash chromatograhpy (hexane/EtOAc), 47% yield; diastereomeric ratio = 90:10; signals for the major isomer: ^1^H NMR (400 MHz, CDCl3): δ_H_ = 7.66–7.60 (m, 1H), 7.55 (dd, *J* = 8.4, 1.1 Hz, 1H), 7.48–7.29 (m, 8H), 5.05 (d, *J* = 5.6 Hz, 1H), 5.01 (s, 1H), 4.36–4.25 (m, 2H), 2.71 (dd, *J* = 12.8, 5.6 Hz, 1H), 2.28 (dd, *J* = 12.8, 10.5 Hz, 1H), 1.46 (s, 3H), 1.35 (td, *J* = 7.1, 4.4 Hz, 3H) ppm; signals for the minor isomer: ^1^H NMR (300 MHz, CDCl3): δH = 7.54–7.48 (m, 2H), 7.44–7.32 (m, 8H), 5.48 (dd, *J* =10.4, 5.5 Hz, 1H), 5.06 (s, 1H), 4.28 (q, *J* = 7.2 Hz, 2H), 2.57 (dd, *J* = 12.4, 5.6 Hz, 1H), 2.49–2.39 (m, 1H), 1.77 (s, 3H), 1.44 (s, 3H) ppm; ^13^C NMR (75 MHz, CDCl3): δ_C_ = 172.2, 145.9, 141.5, 128.7, 128.4, 128.3, 128.0, 127.6, 126.7, 126.7, 126.5, 126.1, 125.8, 86.0, 81.3, 60.8, 51.2, 48.3, 24.7, 14.3 ppm; MS (EI): *m*/*z* 310 (M^+^, 0.59%), 237 (14), 191 (100), 147 (12), 145 (26), 120 (18), 115 (23), 105 (45), 91 (27), 77 (12); HRMS (GC/MS-EI/Q-TOF): *m*/*z* calcd. for C_20_H_22_O_3_ 310.1569, found 310.1565.

Ethyl 3-methyl-3-phenyl-2,3,3a,8b-tetrahydrofuro[3,2-b]benzofuran-2-carboxylate (**3dl**): yellow oil; purification by flash chromatography (hexane/EtOAc), 44% yield; diastereomeric ratio = 70:30; ^1^H NMR (300 MHz, CDCl_3_): δ_H_ = 7.62–7.29 (m, 7H), 7.06–6.80 (m, 2H), 5.70 (d, *J* = 6.2 Hz, 1H), 5.17 (d, *J* = 6.2 Hz, 1H), 4.94 (s, 1H), 4.05 (qd, *J* = 7.1, 1.4 Hz, 2H), 1.56 (s, 3H), 1.07 (t, *J* = 7.1 Hz, 3H) ppm; ^13^C NMR (101 MHz, CDCl_3_): δ_C_ = 169.2, 160.3, 139.0, 131.0, 127.9, 127.9, 126.9, 126.5, 125.0, 121.4, 109.9, 93.5, 81.8, 79.9, 61.1, 54.5, 20.8, 14.0 ppm; MS (EI): *m*/*z* 234 (M^+^, 40%), 251 (30), 221 (23), 208 (16), 207 (100), 178 (13), 145 (16), 133 (43), 118 (23), 105 (92); HRMS (GC/MS-EI/Q-TOF): *m*/*z* calcd. for C_20_H_20_O_4_ 324.1362, found 324.1361.

## 4. Conclusions

In conclusion, we have described a new methodology for the straightforward synthesis of substituted tetrahydrofurans based on the reaction of electron-rich alkenes with epoxides mediated by fluorinated alcohol 1,1,1,3,3,3-hexafluoroisopropanol (HFIP). Although the yields achieved are moderate in most of the cases, the procedure can be envisioned as environmentally benign as it has a perfect atom economy and the reactants are readily available from raw materials such as alkenes with minimum manipulation. Using this methodology, not only densely substituted furans, but also spiro- and polycyclic compounds containing furan moiety were obtained. Additionally, preliminary mechanistic studies point towards a purely ionic pathway (S_N_1-type) or S_N_2-like mechanism depending on the nucleophilicity of the alkene employed.

## Figures and Tables

**Table 1:** Optimization of the reaction parameters ^a^.

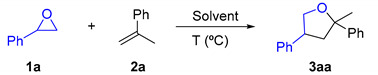			
Entry	Solvent	Temp (°C)	Conv. (%) ^b^
1	H_2_O	45	13
2	*i*PrOH	45	0
3	HFIP	45	72
4	TFE	45	3
5	None	45	2
6	HFIP/CH_2_Cl_2_ (9/1)	45	50
7	HFIP/CH_2_Cl_2_ (1/1)	45	15
8	HFIP/CH_2_Cl_2_ (1/9)	45	23
9	HFIP	25	30
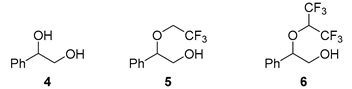			

^a^ All reactions were carried out using 0.15 mmol of **1a** and 0.25 mmol of **2a** in 150 µL of the solvent at 45 °C for 20 h. ^b^ Conversion towards formation of **3aa**, determined by GC-MS.

**Table 2:** Reaction between styrene oxide and electron-rich alkenes ^a^.

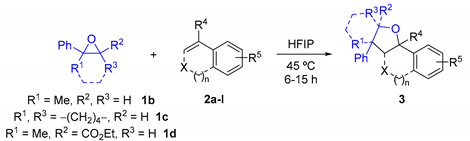
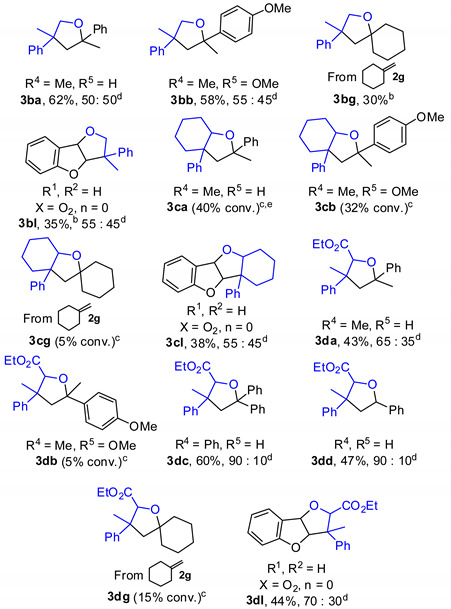

^a^ All reactions were carried out using 0.15 mmol of **1a** and 0.25 mmol of **2a** in 150 µL of the solvent. ^b^ Estimation of the yield from ^1^H NMR data. ^c^ Conversion towards formation of tetrahydrofuran, determined by ^1^H NMR and/or GC-MS. ^d^ Diastereomeric ratio determined by ^1^H NMR and/or GC-MS of the crude compounds. ^e^ Product decomposition after purification was observed.

## Supplementary Materials

The following are available online at https://www.mdpi.com/1420-3049/25/15/3464/s1, Experimental procedures, mechanism elucidation tests, compound characterization data, and copies of NMR spectra for all new compounds.
