# Supplementary material for: HFIP-Promoted Synthesis of Substituted Tetrahydrofurans by Reaction of Epoxides with Electron-Rich Alkenes
Source: Molecules. 2020 Jul 30;25(15):3464. doi: 10.3390/molecules25153464 (PMC7435839; doi:10.3390/molecules25153464)
Supplement: Supplementary file 1 [file molecules-25-03464-s001.pdf]

SUPPORTING INFORMATION

Synthesis of Substituted Tetrahydrofurans through HFIP-Promoted Ring-Opening Reaction of Epoxides with Electron-Rich Alkenes

Natalia Llopis [1] and Alejandro Baeza [1,*]

[1] Departamento de Química Orgánica and Instituto de Síntesis Orgánica (ISO), Facultad de Ciencias, Universidad de Alicante. Apdo. 99, E-03080 Alicante, Spain; natalia.llopis@ua.es.

* Correspondence: alex.baeza@ua.es; Tel.: +34-965-903-728

Mechanism Elucidation Tests and HPLC Chromatograms 2

General Remarks 6

General Procedure and Physical and Spectroscopical Data of Substituted Tetrahydrofurans 7

NMR Spectra of New Isolated Substituted Tetrahydrofurans 12

Mechanism Elucidation Tests and HPLC Chromatograms

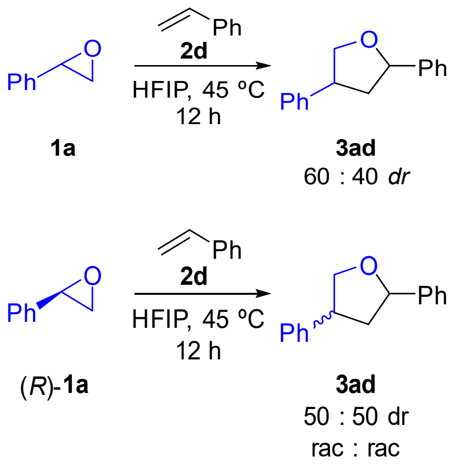

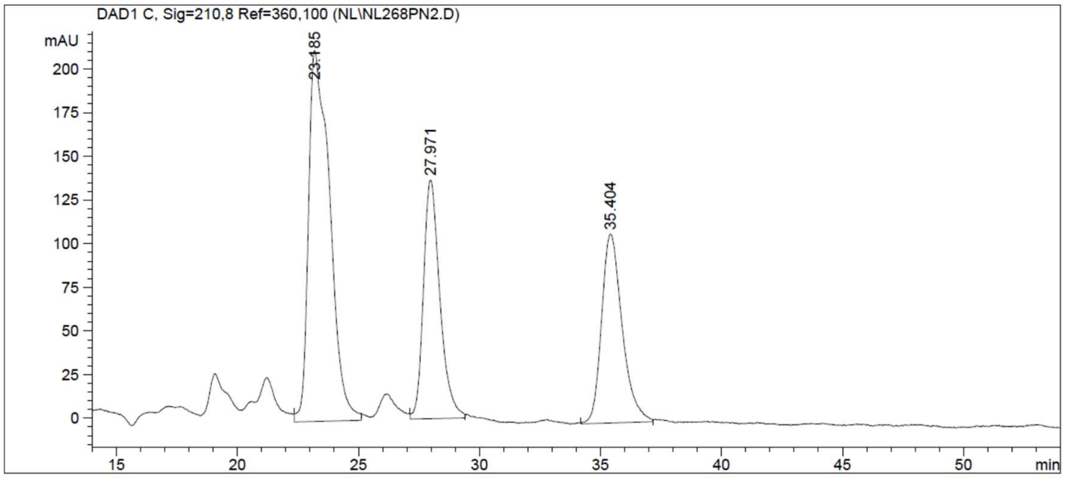


```
Signal 1: DAD1 C, Sig=210,8 Ref=360,100
```

Peak #	RetTime [min]	Type	Width [min]	Area [mAU*s]	Height [mAU]	Area %
1	23.185	VB	0.8018	1.29667e4	212.52806	50.1356
2	27.971	VV	0.6385	6476.85498	136.76041	25.0426
3	35.404	VB	0.7123	6419.72705	108.18962	24.8218

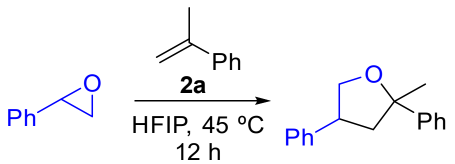

1a **3aa**

60 : 40 *dr*

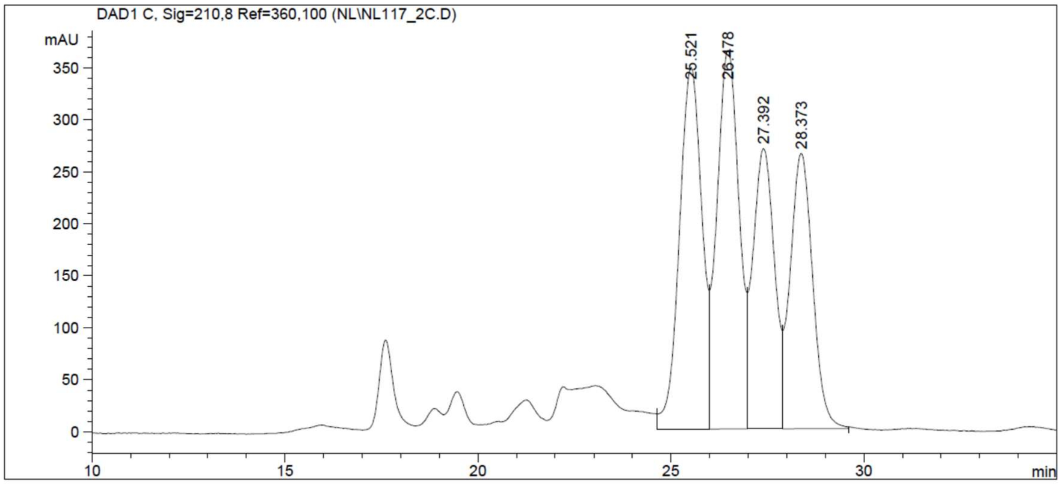

Signal 1: DAD1 C, Sig=210,8 Ref=360,100

Peak #	RetTime [min]	Type	Width [min]	Area [mAU*s]	Height [mAU]	Area %
1	25.521	VV	0.5826	1.40832e4	347.67239	28.2445
2	26.478	VV	0.5894	1.45832e4	364.00748	29.2472
3	27.392	VV	0.5674	1.04316e4	269.58633	20.9210
4	28.373	VV	0.5701	1.07638e4	264.90396	21.5873

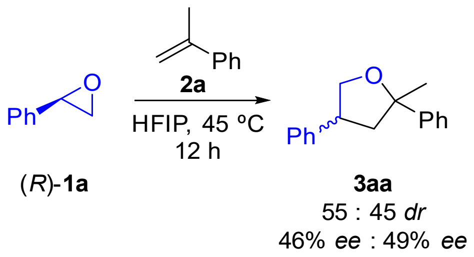

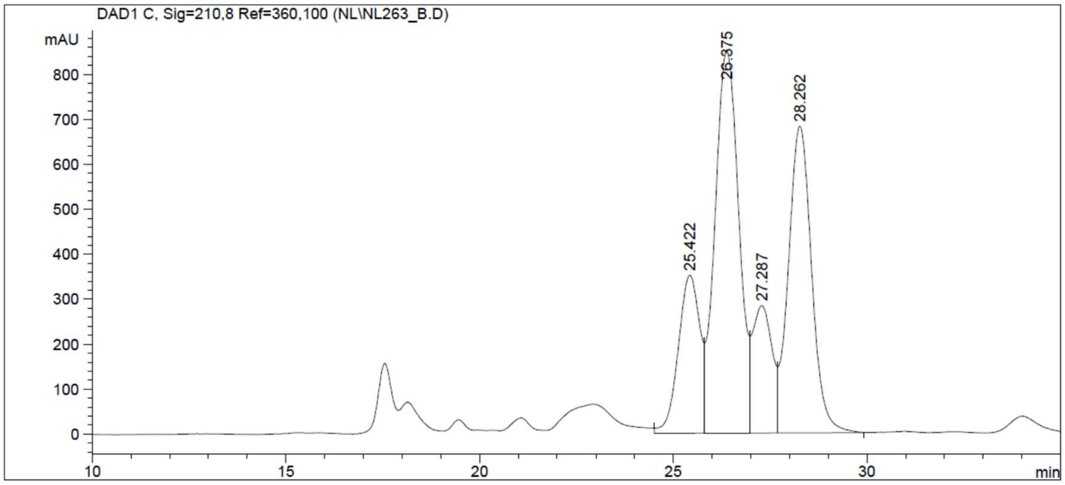

Signal 1: DAD1 C, Sig=210,8 Ref=360,100

Peak #	RetTime [min]	Type	Width [min]	Area [mAU*s]	Height [mAU]	Area %
1	25.422	VV	0.5828	1.35956e4	351.85425	15.3457
2	26.375	VV	0.6061	3.65090e4	851.74103	41.2085
3	27.287	VV	0.5001	9925.68555	283.78348	11.2033
4	28.262	VB	0.6265	2.85655e4	682.18329	32.2425

General Remarks:

All reagents and solvents were obtained commercially and used without further purification. Substrates that were not commercially available were synthesized according to known literature procedures.

NMR spectra were performed on a Bruker AV-300 or Bruker AV-400 (Bruker Corporation) using $CDCl_3$ as solvent and TMS as internal standard unless otherwise stated.

Low resolution mass spectra (MS) were recorded in the electron impact mode (EI, 70 eV, He as carrier phase) using an Agilent GC/MS 5973 Network Mass Selective Detector spectrometer apparatus equipped with a HP-5MS column (Agilent technologies, 30 m × 0.25 mm) and giving fragment ions in m/z with relative intensities (%) in parentheses. Low-resolution electron impact (EI) mass spectra were obtained at 70eV on Agilent GC/MS-5973N apparatus equipped with a HP-5MS column (Agilent technologies, 30 m × 0.25 mm). High-resolution mass spectra (HRMS) were obtained on an Agilent 7200 Quadrupole-Time of Flight apparatus (Q-TOF), the ionization employed being electron impact (EI).

Chiral HPLC analysis was performed in an Agilent 1100 Series HPLC equipped with a G1315B diode array detector and a Quat Pump G1311A equipped with the corresponding Daicel chiral column.

Analytical TLC was performed on Merck silica gel plates and the spots visualized with UV light at 254 nm. Flash chromatography employed Merck silica gel 60 (0.040-0.063 mm). Silica gel 60 F_{254} containing gypsum was employed for preparative layer chromatography.

General procedure for the HFIP-promoted synthesis of substituted tetrahydrofurans.

In a capped tube, onto a mixture of the corresponding epoxide (0.15 mmol) and alkene (0.25 mmol), HFIP (150 μL) was added in one portion. The reaction was then stirred at 45 ºC for 6-15 hours, until the reaction was judged to be completed (no starting epoxide remaining) by GC-MS. After this time, solvent was evaporated and the crude material was directly purified by flash chromatography or preparative TLC.

Physical and Spectroscopical Data of Substituted Tetrahydrofurans

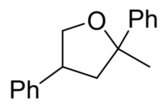

3aa

2-Methyl-2,4-diphenyltetrahydrofuran (3aa):[1] yellow oil; purification by flash chromatography (hexane/EtOAc), 54% yield; (*cis/trans*) = 55:45; ^{1}H NMR (300 MHz, $CDCl_3$): *cis* isomer: δ_H = 7.52 – 7.45 (m, 4H), 7.43 – 7.28 (m, 10H), 7.27 – 7.16 (m, 6H), 4.42 (t, *J* = 7.6 Hz, 1H), 3.84 (dd, *J* = 10.0, 8.2 Hz, 1H), 3.70 (tt, *J* = 10.3, 7.7 Hz, 1H), 2.41 – 2.15 (m, 2H), 1.63 (s, 3H) ppm; further signals for the *trans* isomer: δ_H = 4.35 (t, *J* = 8.4 Hz, 1H), 4.00 (t, *J* = 8.7 Hz, 1H), 3.31 (ddd, *J* = 15.9, 11.3, 8.6 Hz, 1H), 2.81 – 2.60 (m, 2H), 1.68 (s, 3H) ppm; ^{13}C NMR (101 MHz, $CDCl_3$): *cis* isomer: δ_C = 148.9, 140.8, 128.5, 128.3, 127.4, 126.6, 126.5, 124.5, 74.4, 47.9, 45.8, 30.6, ppm; further signals for *trans* isomer: 147.6, 141.6, 128.5, 128.2, 127.3, 126.6, 126.4, 124.7, 73.9, 48.3, 44.6, 30.2 ppm; MS (EI): *m/z* 238 (M^+, 0.31%), 224 (19), 223 (100), 193 (12), 117 (27), 115 (18), 105 (90), 91 (16), 77 (18). Chiral HPLC analysis: Chiralpak IA column, Hexane/iPrOH 99:1, flow rate = 0.2 mL/min, λ = 210 nm, retention times: = 25.5 and 26.5 min. (major diastereosiomer) and 27.4 and 28.4 min. (minor diastereoisomer).

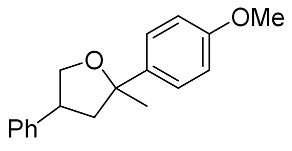

3ab

2-(4-Methoxyphenyl)-2-methyl-4-phenyl tetrahydrofuran (3ab): orange oil; purification by flash chromatography (hexane/EtOAc), 67% yield; (*cis/trans*) = 55:45; ^{1}H NMR (300 MHz, $CDCl_3$): *cis* isomer: δ_H = 7.43 – 7.37 (m, 3H), 7.33 – 7.28 (m, 3H), 7.27 – 7.16 (m, 6H), 6.95 – 6.93 (m, 2H), 6.92 – 6.90 (m, 2H), 4.40 (t, *J* = 7.9 Hz, 1H), 3.84 (s, 3H), 3.82 (dd, *J* = 3.5, 1.9 Hz, 1H), 3.76 – 3.64 (m, 1H), 2.63 (dd, *J* = 12.4, 8.0 Hz, 1H), 2.32 (dd, *J* = 12.3, 10.5 Hz, 1H), 2.24 – 2.17 (m, 1H), 1.61 (s, 3H) ppm; further signals for the *trans* isomer: δ_H = 4.33 (t, *J* = 8.4 Hz, 1H), 3.98 (t, *J* = 8.6 Hz, 1H), 3.85 (s, 3H), 3.39 – 3.25 (m, 1H), 2.71 (dd, *J* = 12.1, 7.1 Hz, 1H), 2.32 (dd, *J* = 12.3, 10.5 Hz, 1H), 1.66 (s, 3H)

[1] G. Hilt, P. Bolze and K. Harms, *Chem. Eur. J.* 2007, **13**, 4312.

ppm; ^{13}C NMR (101 MHz, $CDCl_3$): mixture of isomers, δ_C = 158.3, 158.2, 141.7, 141.1, 141.0, 139.7, 128.5, 128.5, 127.4, 127.3, 126.6, 126.6, 125.8, 125.7, 113.6, 113.5, 85.2, 84.7, 74.4, 73.9, 55.3, 55.2, 48.3, 48.1, 45.8, 44.6, 30.6, 30.3 ppm; MS (EI): *m/z* 268 (M^+, 6%), 254 (17), 253 (93), 135 (100), 117 (14), 91 (11); HRMS (GC/MS-EI/Q-TOF): *m/z* calcd. for $C_{18}H_{20}O_2$ 268.1463, found 268.1463.

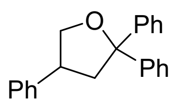

3ac

2,2,4-Triphenyltetrahydrofuran (3ac): yellow oil; purification by flash chromatography (hexane/EtOAc), 38% estimated yield (not purely isolated); ^{1}H NMR (300 MHz, $CDCl_3$): δ_H = 7.54 – 7.49 (m, 4H), 7.37 – 7.34 (m, 4H), 7.34 – 7.30 (m, 4H), 7.27 – 7.24 (m, *J* = 1.6 Hz, 2H), 7.24 – 7.22 (m, 1H), 4.48 (t, *J* = 8.4 Hz, 1H), 4.07 (t, *J* = 8.7 Hz, 1H), 3.61 – 3.43 (m, *J* = 16.0, 11.0, 5.2 Hz, 1H), 3.23 (dd, *J* = 12.3, 7.1 Hz, 1H) ppm; MS (EI): *m/z* 300 (M^+, 55%), 270 (15), 224 (71), 223 (100), 192 (21), 191 (13), 179 (13), 178 (15), 165 (21), 118 (34), 117 (42), 115 (12), 105 (96), 91 (14), 77 (27); HRMS (GC/MS-EI/Q-TOF): *m/z* calcd. for $C_{22}H_{20}O$ 300.1514, found 300.1509.

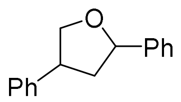

3ad

2,4-Diphenyltetrahydrofuran (Caloxylane A and B) (3ad):1 yellow oil; purification by flash chromatography (hexane/EtOAc), 39% yield; (*cis/trans*) = 65:35; ^{1}H NMR (300 MHz, $CDCl_3$): *cis* isomer: δ_H = 7.49 – 7.29 (m, 20H), 5.11 (dd, *J* = 10.2, 5.7 Hz, 1H), 4.39 (t, *J* = 8.2 Hz, 1H), 4.05 (t, *J* = 8.5 Hz, 1H), 3.74 – 3.63 (m, 1H), 2.85 – 2.72 (m, 1H), 2.05 (q, *J* = 10.5, 1.9 Hz, 1H) ppm; further signals for the *trans* isomer: δ_H = 5.26 (dd, *J* = 7.7, 5.8 Hz, 1H), 4.50 (t, *J* = 8.5, 7.4 Hz, 1H), 3.98 (t, *J* = 8.2 Hz, 1H), 3.63 – 3.51 (m, 1H), 2.57 – 2.45 (m, 1H), 2.36 (q, *J* = 12.5, 8.3, 5.8 Hz, 1H) ppm; ^{13}C NMR (75 MHz, $CDCl_3$): *cis* isomer: δ_C = 142.6, 141.7, 128.6, 128.4, 127.4, 127.2, 125.7, 81.8, 75.1, 46.0, 43.7, pm; further signals for the *trans* isomer: δ_C = 143.6, 142.0, 128.6, 128.3, 127.3, 127.1, 125.5, 80.6, 75.1, 44.4, 42.7 ppm; MS (EI): *m/z* 224 (M^+, 34%), 195 (14), 194 (93), 193 (100), 179 (58), 178 (89), 165 (13), 146 (27), 133 (34), 120 (27), 117 (90), 115 (57), 105 (45), 91 (48), 77 (30). Chiral HPLC analysis: Chiralcel OD-H column, Hexane/iPrOH 99:1, flow rate = 0.7 mL/min, λ = 210 nm, retention times: = 23.1 and 23.2 min. (major diastereosiomer) and 28.0 and 35.4 min. (minor diastereoisomer).

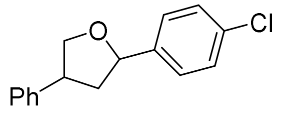

3ae

2-(4-Chlorophenyl)-4-phenyltetrahydrofuran (3ae):1 yellow oil; purification by flash chromatography (hexane/EtOAc), 36% yield; (*cis*/*trans*) = 60:40; ^{1}H NMR (400 MHz, $CDCl_3$): *cis* isomer: δ_H = 7.40 – 7.30 (m, 9H), 5.07 (dd, *J* = 10.1, 5.8 Hz, 1H), 4.38 (t, *J* = 8.2 Hz, 1H), 4.03 (t, *J* = 8.5 Hz, 1H), 3.78 – 3.61 (m, 1H), 2.86 – 2.72 (m, 1H), 1.98 (dd, *J* = 12.4, 10.4 Hz, 1H) ppm; further signals for the *trans* isomer: δ_H = 5.22 (dd, *J* = 7.7, 5.9 Hz, 1H), 4.47

(dd, J = 8.4, 7.5 Hz, 1H), 3.97 (t, J = 8.2 Hz, 1H), 3.54 (t, J = 7.6 Hz, 1H), 2.50 (dt, J = 12.6, 7.7 Hz, 1H), 2.35 – 2.24 (m, 1H) ppm; ^{13}C NMR (101 MHz, $CDCl_3$): *cis* isomer: δ_C = 141.5, 141.2, 133.0, 129.9, 128.8, 128.6, 128.3, 128.2, 127.3, 127.1, 126.7, 81.1, 75.1, 45.9, 43.8 ppm; further signals for the *trans* isomer: δ_C = 142.1, 142.7, 132.8, 129.8, 128.7, 128.5, 128.3, 128.1, 127.2, 126.9, 126.7, 126.4, 79.9, 72.7, 44.3, 42.7 ppm; MS (EI): *m/z* 258 (M^+, 18%), 228 (25), 193 (100), 180 (15), 178 (22), 167 (22), 154 (16), 139 (27), 117 (64), 115 (54), 104 (17), 91 (27), 77 (13).

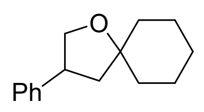

3ag

3-Phenyl-1-oxaspiro[4.5]decane (3ag): yellow solid, purification by flash chromatography (hexane/EtOAc), 45% yield; ^1H NMR (400 MHz, $CDCl_3$): δ_H = 7.36 – 7.31 (m, 3H), 7.27 – 7.23 (m, 2H), 4.23 (t, J = 8.0 Hz, 1H), 3.80 (t, J = 17.6 Hz, 1H), 3.51 (tt, J = 17.6, 8.8 Hz, 1H), 2.30 (dd, J = 12.4, 8.2 Hz, 1H), 1.78 (dd, J = 12.4, 10.5 Hz, 1H), 1.74 – 1.48 (m, 10H) ppm; ^{13}C NMR (126 MHz, $CDCl_3$): δ_C = 141.9, 128.6, 128.5, 128.1, 127.3, 126.5, 83.3, 72.9, 45.0, 38.3, 37.3, 25.61, 23.8, 23.8 ppm; MS (EI): *m/z* 216 (M^+, 55%), 174 (25), 173 (100), 160 (40), 118 (18), 117 (28), 104 (41), 91 (26), 55 (73); HRMS (GC/MS-EI/Q-TOF): *m/z* calcd. for $C_{15}H_{20}O$ 216.1514, found 216.1514.

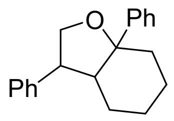

3ai

3,7a-Diphenyloctahydrobenzofuran (3ai): orange oil; purification by flash chromatography (hexane/EtOAc); 34% yield; 55:45 diastereomeric ratio; ^1H NMR (300 MHz, $CDCl_3$): major isomer: δ_H = 7.58 (dd, J = 8.5, 1.1 Hz, 2H), 7.54 – 7.48 (m, 2H), 7.44 – 7.35 (m, 6H), 7.34 – 7.28 (m, 4H), 7.23 (m, 6H), 7.16 – 7.08 (m, 2H), 4.40 (m, 2H), 4.00 – 3.91 (m, 1H), 3.59 (m, 1H), 2.67 (dd, J = 12.0, 5.5 Hz, 1H), 2.13 – 1.85 (m, 4H), 1.83 – 1.52 (m, 12H) ppm; furher signals for the minor isomer: δ_H = 4.27 (t, J = 8.6 Hz, 1H), 3.42 (td, J = 9.6, 5.5 Hz, 1H), 2.57 (dt, J = 11.4, 5.7 Hz, 1H) ppm; ^{13}C NMR (75 MHz, $CDCl_3$): *major* isomer: δ_C = 148.6, 141.2, 128.6, 128.2, 128.1, 126.9, 126.4, 125.9, 84.8, 72.6, 51.2, 46.6, 35.8, 24.2, 21.9, 20.0 ppm; further signals for *minor* isomer: δ_C = 146.5, 138.2, 128.3, 128.1, 127.9, 126.7, 126.2, 124.5, 86.8, 67.3, 49.4, 46.4, 38.3, 24.6, 22.1, 20.0 ppm; MS (EI): *m/z* 278 (M^+, 85%), 236 (19), 235 (100), 221 (18), 115 (14), 105 (67), 91 (25), 77 (18); HRMS (GC/MS-EI/Q-TOF): *m/z* calcd. for $C_{20}H_{22}O$ 278.1671, found 278.1667.

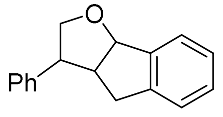

3aj

3-Phenyl-3, 3a, 4, 8b-tetrahydro-2H-indeno [1,2-b]furan (3aj):1 yellow oil; purification by flash chromatography (hexane/EtOAc), 40% yield; (*cis*/*trans*) = 60:40; ^1H NMR (300 MHz, $CDCl_3$): *cis* isomer: δ_H = 7.53 – 7.47 (m, 2H), 7.39 – 7.29 (m, 8H), 5.73 (d, J = 7.2 Hz, 1H), 4.13 (dd, J = 8.6, 6.7 Hz, 1H),

3.92 (dd, J = 8.6, 7.7 Hz, 1H), 3.26 – 3.16 (m, 2H), 3.12 – 3.03 (m, 1H), 3.01 – 2.91 (m, 1H) ppm; further signals for the *trans* isomer: δ_H = 7.51 – 7.46 (m, 1H), 7.36 – 7.24 (m, 6H), 7.14 – 7.09 (m, 2H), 5.69 (d, J = 6.8 Hz, 1H), 4.27 – 4.21 (m, 1H), 3.82 – 3.73 (m, 2H), 3.59 – 3.47 (m, 1H), 2.83 (dd, J = 17.4, 9.3 Hz, 1H), 2.60 (dd, J = 17.4, 4.8 Hz, 1H) ppm; ^{13}C NMR (75 MHz, $CDCl_3$) : mixture of isomers, δ_C = 142.5, 141.9, 141.7, 141.6, 128.8, 128.7, 128.4, 128.4, 127.5, 127.2, 127.0, 126.7, 126.5, 125.6, 125.2, 124.4, 87.9, 87.8, 74.6, 68.2, 53.3, 50.1, 48.5, 45.5, 38.7, 36.6 ppm; MS (EI): *m/z* 236 (M^+, 34%), 207 (17), 206 (100), 205 (27), 128 (20), 115 (27), 91 (86).

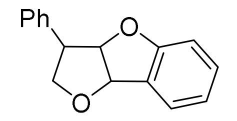

3al

3-Phenyl-2,3,3a,8b-tetrahydrofuro[3,2-b]benzofuran (3al): yellow oil; purification by flash chromatography (hexane/EtOAc), 40% estimated yield, mixture of isomers (not purely isolated); ^{1}H NMR (400 MHz, $CDCl_3$): δ_H = 7.50 – 7.46 (m, 2H), 7.40 – 7.38 (m, 2H), 7.37 – 7.36 (m, 3H), 7.36 – 7.31 (m, 6H), 7.31 – 7.27 (m, 3H), 7.00 (tdd, J = 7.4, 1.9, 0.9 Hz, 2H), 6.90 (t, J = 5.6 Hz, 1H), 6.85 (d, J = 8.1 Hz, 1H), 5.87 (d, J = 5.7 Hz, 1H), 5.82 (d, J = 6.0 Hz, 1H), 5.32 (t, J = 5.6 Hz, 1H), 5.20 (dd, J = 6.0, 1.2 Hz, 1H), 4.26 – 4.19 (m, 1H), 4.10 (d, J = 2.4 Hz, 1H), 4.08 (d, J = 2.4 Hz, 1H), 3.96 (dd, J = 9.2, 5.5 Hz, 1H), 3.75 – 3.70 (m, 1H), 3.68 (d, J = 4.7 Hz, 1H). ppm; MS (EI): *m/z* 238 (M^+, 34%), 220 (66), 219 (43), 208 (45), 207 (100), 191 (15), 189 (17), 178 (19), 165 (13), 131 (24), 117 (12); HRMS (GC/MS-EI/Q-TOF): *m/z* calcd. for $C_{16}H_{14}O$ 238.0994, found 238.0990.

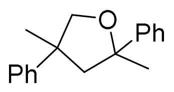

3ba

2,4 –Dimethyl – 2,4 – diphenyltetrahydrofuran (3ba):[2] yellow solid; purification by flash chromatography (hexane/EtOAc), 62% yield; (*cis/trans*) = 50:50; the *cis* isomer is highlighted in bold; ^{1}H NMR (300 MHz, $CDCl_3$): δ_H = 7.54 – 7.49 (m, 2H), 7.45 – 7.39 (m, 2H), 7.39 – 7.30 (m, 8H), 7.26 – 7.21 (m, 4H), 7.20 – 7.10 (m, 4H), **4.30 (d, *J* = 8.6 Hz, 1H),** 4.10 (dd, J = 8.4, 1.0 Hz, 1H), **4.06 (d, *J* = 8.5 Hz, 1H)**, 3.96 (dd, J = 8.4, 0.6 Hz, 1H), 2.72 (d, J = 12.6 Hz, 1H), **2.58 (s, 2H)**, 2.47 (dd, J = 12.6, 1.1 Hz, 1H), 1.70 (s, 3H), **1.59 (s, 6H)**, 1.50 (s, 3H) ppm; ^{13}C NMR (101 MHz, $CDCl_3$): mixture of isomers, δ_C = 149.5, 149.3, 148.2, 147.7, 128.4, 128.3, 128.2, 128.1, 126.3, 126.2, 126.0, 126.0, 125.9, 125.8, 125.7, 124.5, 124.4, 124.4, 84.8, 84.7, 78.0, 77.8, 53.9, 53.7, 48.9, 48.4, 32.7, 31.6, 29.9, 28.5 ppm; MS (EI): *m/z* 252(M^+, 0.08%), 237 (100), 207 (12), 129 (13), 117 (29), 105 (97), 91 (14), 77 (15).

[2] S. Sultana, N. R. Devi and A. K. Saikia, *Asian J. Org. Chem*. 2015, **4** ,1281–1288

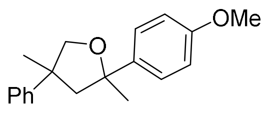

3bb

2–(4–Methoxyphenyl)–2,4–dimethyl-4-phenyl tetrahydrofuran (3bb): white solid; purification by flash chromatography (hexane/EtOAc), 58% yield; (*cis/trans*) = 45:55; the cis isomer is highlighted in bold; ^{1}H NMR (300 MHz, $CDCl_3$): δ_H = 7.36 – 7.30 (m, 3H), 7.27 – 7.22 (m, 2H), 7.16 – 7.09 (m, 2H), 6.87 – 6.82 (m, 2H), **4.28 (d, J = 8.6 Hz, 1H)**, 4.07 (d, J = 9.1 Hz, 1H), **4.03 (s, 1H)**, 3.95 (d, J = 8.4 Hz, 1H), **3.84 (s, 3H)**, 3.80 (s, 3H), 2.70 (d, J = 12.6 Hz, 1H), **2.55 (d, J = 2.2 Hz, 1H)**, 2.42 (d, J = 12.5 Hz, 1H), 1.67 (s, 3H), 1.58 (s, 3H), **1.48 (s, 3H)**, **1.23 (s, 3H)** ppm; ^{13}C NMR (101 MHz, $CDCl_3$): only major isomer is given, δ_c = 157.9, 147.7, 141.6, 128.3, 126.0, 125.9, 125.6, 113.5, 84.6, 77.8, 55.3, 53.9, 48.9, 32.8, 29.9 ppm; MS (EI): *m/z* 282(M^+, 9%), 268 (16), 267 (83), 135 (100), 117 (13); HRMS (GC/MS-EI/Q-TOF): *m/z* calcd. for $C_{19}H_{22}O_2$ 282.1620, found 282.1620.

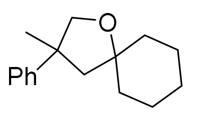

3bg

3-Methyl-3-phenyl-1-oxaspiro[4.5]decane (3bg): colourless oil; purification by flash chromatography (hexane/EtOAc), 30% estimated yield (not purely isolated); ^{1}H NMR (300 MHz, $CDCl_3$): δ_H = 7.36 – 7.30 (m, 4H), 7.26 – 7.23 (m, 1H), 4.05 (d, *J* = 8.7 Hz, 1H), 3.94 (d, *J* = 8.5 Hz, 1H), 2.12 (d, *J* = 12.6 Hz, 1H), 1.98 (dd, *J* = 12.6, 0.7 Hz, 1H), 1.79 – 1.7 (m, 5H), 1.61 (d, *J* = 2.7 Hz, 2H) 1.51 (d, *J* = 6.0 Hz, 3H), 1.46 (s, 3H) ppm; MS (EI): *m/z* 230(M^+, 43%), 216 (16), 215 (100), 187 (59), 118 (19), 117 (25), 91 (14), 55 (30); HRMS (GC/MS-EI/Q-TOF): *m/z* calcd. for $C_{16}H_{22}O$ 230.1671, found 230.1672.

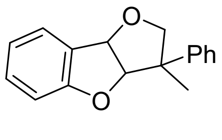

3bl

3-Methyl-3-phenyl-2,3,3a,8b-tetrahydrofuro [3,2-b] benzofuran (3bl): Inseparable mixture of regioisomers. The major isomer data is highlighted in bold. Yellow solid; purification by flash chromatography (hexane/EtOAc), 35% estimated yield (not purely isolated); ^{1}H NMR (400 MHz, $CDCl_3$): δ_H = 7.60 – 7.49 (m, 3H), 7.48 – 7.38 (m, 3H), 7.38 – 7.30 (m, 8H), **7.27 – 7.21 (m, 2H)**, 7.00 – 6.88 (m,2H), 5.60 (d, *J* = 6.0 Hz, 1H), **5.15 (d, *J* = 6.0 Hz, 1H)**, 4.31 (d, *J* = 9.3 Hz, 1H), **4.19 (d, *J* = 12.4 Hz, 1H)**, 3.91 (d, *J* = 5.7 Hz, 1H), **3.82 (d, *J* = 12.2 Hz, 1H)**, 3.64 (d, *J* = 9.3 Hz, 1H), **3.60 (d, *J* = 12.4 Hz, 1H)**, 1.61 (s, 3H), **1.52 (s, 3H)**, ppm; MS (EI): *m/z* 252 (M^+, 71%), 237 (14), 221 (15), 207 (100), 194 (20), 178 (15), 145 (23), 131 (41), 129 (11), 118 (36), 115 (16), 105 (14), 91 (20), 89(15), 77 (14); HRMS (GC/MS-EI/Q-TOF): *m/z* calcd. for $C_{17}H_{16}O_2$ 252.1150, found 252.1149.

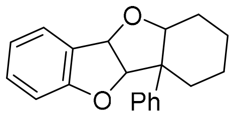

3cl

4a-Phenyl-1,2,3,4,4a,4b,9b,10a-octahydrobenzofuro[3,2-b]benzofuran (3cl): white solid; purification by flash chromatography (hexane/EtOAc), 38% yield; 55:45 mixture of diastereoisomers; ^{1}H NMR (400 MHz, $CDCl_3$): δ_H = 7.53 –

7.47 (m, 3H), 7.45 – 7.34 (m, 3H), 7.34 – 7.30 (m, 1H), 7.02 – 6.90 (m, 2H), 5.54 (d, *J* = 7.8 Hz, 1H), 5.15 (d, *J* = 7.9 Hz, 1H), 4.43 (t, *J* = 2.5 Hz, 1H), 2.17 – 2.03 (m, 1H), 2.00 – 1.85 (m, 1H), 1.51 – 1.39 (m, 3H), 1.05 – 0.82(m, 3H) ppm; ^{13}C NMR (400 MHz, $CDCl_3$): δ_C =130.7, 130.4, 128.5, 128.2, 128.0, 127.0, 126.4, 126.3, 126.2, 126.0, 120.9, 120.7, 110.0, 109.7, 96.8, 93.9, 80.7, 80.5, 78.9, 77.2, 75.3, 32.2, 29.7, 28.7, 26.5, 25.1, 21.1, 20.4, 20.2 ppm; MS (EI): *m/z* 292 (M^+, 17%), 274 (20), 208 (17), 207 (100), 194 (25), 91 (11); HRMS (GC/MS-EI/Q-TOF): *m/z* calcd. for $C_{20}H_{20}O_2$ 292.1463, found 292.1460.

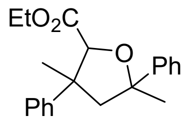

3da

Ethyl 3,5-dimethyl-3,5-diphenyltetrahydrofuran-2-carboxylate (3da): yellow solid; purification by flash chromatography (hexane/EtOAc), 43% yield; 65:35 mixture of diastereoisomers; ^{1}H NMR (300 MHz, $CDCl_3$): δ_H = 7.68 – 7.62 (m, 2H), 7.54 – 7.49 (m, 2H), 7.42 – 7.34 (m, 6H), 7.26 (d, *J* = 2.0 Hz, 1H), 5.08 (s, 1H), 4.21 (qd, *J* = 7.1, 1.5 Hz, 2H), 2.75 (d, *J* = 12.9 Hz, 1H), 2.65 (d, *J* = 12.8 Hz, 1H), 1.61 (s, 3H), 1.23 (t, *J* = 7.1 Hz, 3H) 1.22 (t, *J* = 7.1 Hz, 3H) ppm; ^{13}C NMR (101 MHz, $CDCl_3$): only major isomer is given, δ_C = 170.6, 148.7, 145.4, 128.3, 128.1, 126.4, 126.3, 126.3, 124.6, 84.7, 84.3, 60.7, 56.6, 31.8, 29.7, 23.5, 14.2 ppm; MS (EI): *m/z* 324 (M^+, 0.13%), 309 (86), 251 (48), 233 (18), 207 (27), 173 (14), 133 (17), 129 (16), 105 (100), 91 (15), 77 (15); HRMS (GC/MS-EI/Q-TOF): *m/z* calcd. for $C_{21}H_{24}O_3$ 324.1725, found 324.1715.

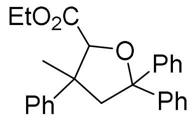

3dc

Ethyl 3-methyl-3,5,5-triphenyltetrahydrofuran-2-carboxylate (3dc): yellow oil; purification by flash chromatography (hexane/EtOAc), 60% yield; 90:10 mixture of diastereoisomers; ^{1}H NMR (300 MHz, $CDCl_3$): δ_H = 7.70 – 7.64 (m, 3H), 7.52 – 7.48 (m, 3H), 7.39 – 7.30 (m, 6H), 7.24 – 7.18 (m, 3H), 4.96 (s, 1H), 4.14 (q, *J* = 7.1 Hz, 2H), 3.33 (d, *J* = 13.1 Hz, 1H), 3.01 (d, *J* = 13.0 Hz, 1H), 1.34 (s, 3H), 1.20 (s, 3H) ppm; ^{13}C NMR (101 MHz, $CDCl_3$): δ_C = 170.3, 147.2, 147.0, 145.1, 128.5, 128.3, 128.2, 128.1, 127.8, 126.8, 126.6, 126.5, 126.4, 126.3, 125.7, 125.5, 125.1, 87.6, 85.1, 60.7, 56.0, 51.2, 23.9, 14.2 ppm; MS (EI): *m/z* 386 (M^+, 0.75%), 314 (15), 313 (59), 309 (45), 295 (24), 269 (26), 206 (12), 196 (75), 191 (30), 181 (12), 178 (16), 167 (86), 165 (30), 133 (24), 105 (100), 91 (1), 77 (19); HRMS (GC/MS-EI/Q-TOF): *m/z* calcd. for $C_{26}H_{26}O_3$ 386.1882, found 386.1884.

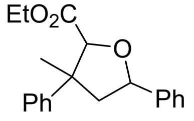

3dd

Ethyl-3-methyl-3,5-diphenyltetrahydrofuran-2-carboxylate (3dd): orange solid; purification by flash chromatograhpy (hexane/EtOAc), 47% yield; a mixture of diastereoisomers was obtained, 90:10 mixture of diastereoisomers; the signals for the major isomer: ^{1}H NMR (400 MHz, $CDCl_3$): δ_H = 7.66 – 7.60 (m,

1H), 7.55 (dd, J = 8.4, 1.1 Hz, 1H), 7.48 – 7.29 (m, 8H), 5.05 (d, J = 5.6 Hz, 1H), 5.01 (s, 1H), 4.36 – 4.25 (m, 2H), 2.71 (dd, J = 12.8, 5.6 Hz, 1H), 2.28 (dd, J = 12.8, 10.5 Hz, 1H), 1.46 (s, 3H), 1.35 (td, J = 7.1, 4.4 Hz, 3H) ppm; the signals for the minor isomer: ^{1}H NMR (300 MHz, $CDCl_3$): δ_H = 7.54 – 7.48 (m, 2H), 7.44 – 7.32 (m, 8H), 5.48 (dd, J =10.4, 5.5 Hz, 1H), 5.06 (s, 1H), 4.28 (q, J = 7.2 Hz, 2H), 2.57 (dd, J = 12.4, 5.6 Hz, 1H), 2.49 – 2.39 (m, 1H), 1.77 (s, 3H), 1.44 (s, 3H) ppm ; ^{13}C NMR (75 MHz, $CDCl_3$): δ_C = 172.2, 145.9, 141.5, 128.7, 128.4, 128.3, 128.0, 127.6, 126.7, 126.7, 126.5, 126.1, 125.8, 86.0, 81.3, 60.8, 51.2, 48.3, 24.7, 14.3 ppm; MS (EI): *m/z* 310 (M^+, 0.59%), 237 (14), 191 (100), 147 (12), 145 (26), 120 (18), 115 (23), 105 (45), 91 (27), 77 (12); HRMS (GC/MS-EI/Q-TOF): *m/z* calcd. for $C_{20}H_{22}O_3$ 310.1569, found 310.1565.

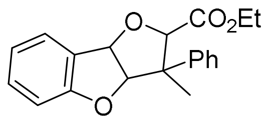

3dl

Ethyl 3-methyl-3-phenyl-2,3,3a,8b-tetrahydrofuro[3,2-b]benzofuran-2-carboxylate (3dl): yellow oil; purification by flash chromatography (hexane/EtOAc), 44% yield; 70:30 mixture of diastereoisomers; ^{1}H NMR (300 MHz, $CDCl_3$): δ_H = 7.62 – 7.29 (m, 7H), 7.06 – 6.80 (m, 2H), 5.70 (d, J = 6.2 Hz, 1H), 5.17 (d, J = 6.2 Hz, 1H), 4.94 (s, 1H), 4.05 (qd, J = 7.1, 1.4 Hz, 2H), 1.56 (s, 3H), 1.07 (t, J = 7.1 Hz, 3H) ppm; ^{13}C NMR (101 MHz, $CDCl_3$): δ_C = 169.2, 160.3, 139.0, 131.0, 127.9, 127.9, 126.9, 126.5, 125.0, 121.4, 109.9, 93.5, 81.8, 79.9, 61.1, 54.5, 20.8, 14.0 ppm; MS (EI): *m/z* 324 (M^+, 40%), 251 (30), 221 (23), 208 (16), 207 (100), 178 (13), 145 (16), 133 (43), 118 (23), 105 (92); HRMS (GC/MS-EI/Q-TOF): *m/z* calcd. for $C_{20}H_{20}O_4$ 324.1362, found 324.1361.

NMR Spectra of New Isolated Substituted Tetrahydrofurans

2-(4-methoxyphenyl)-2-methyl-4-phenyl tetrahydrofuran (3ab) [*cis* isomer]

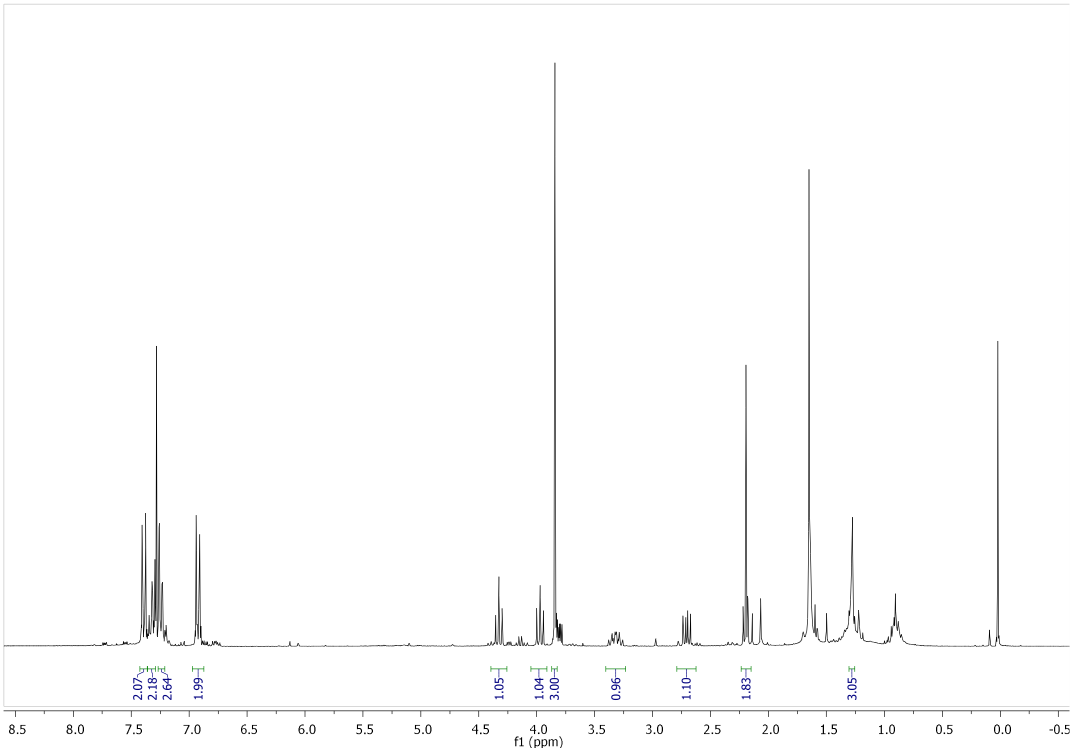

2-(4-methoxyphenyl)-2-methyl-4-phenyl tetrahydrofuran (3ab) [*cis/trans* mixture]

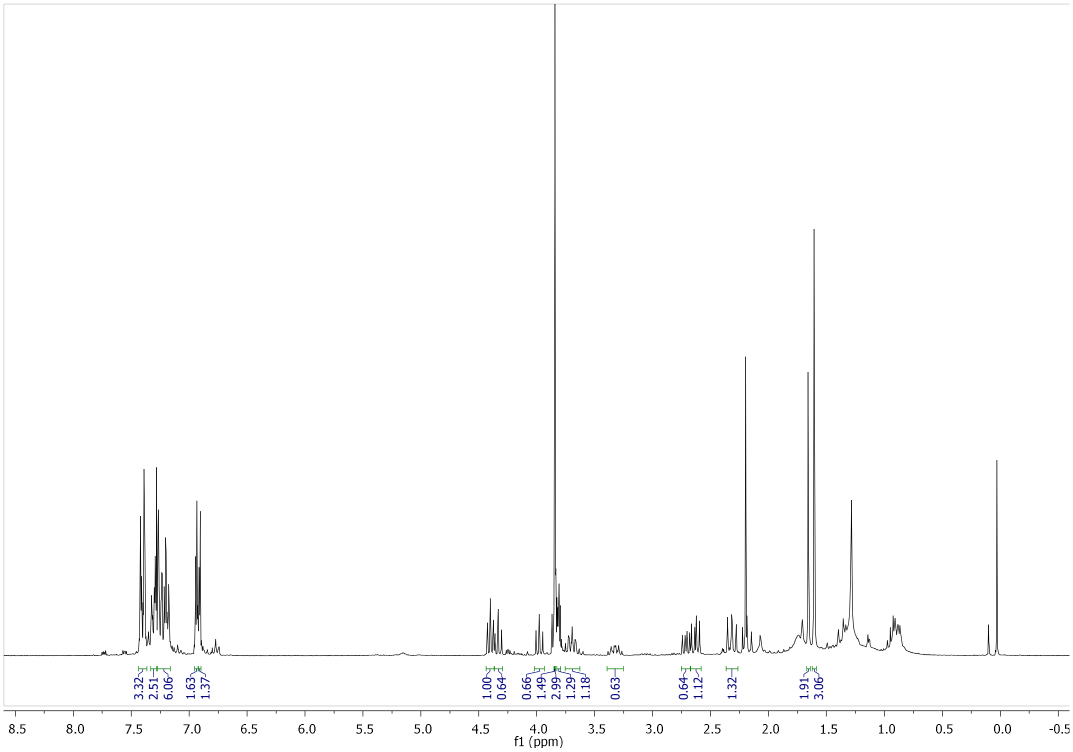

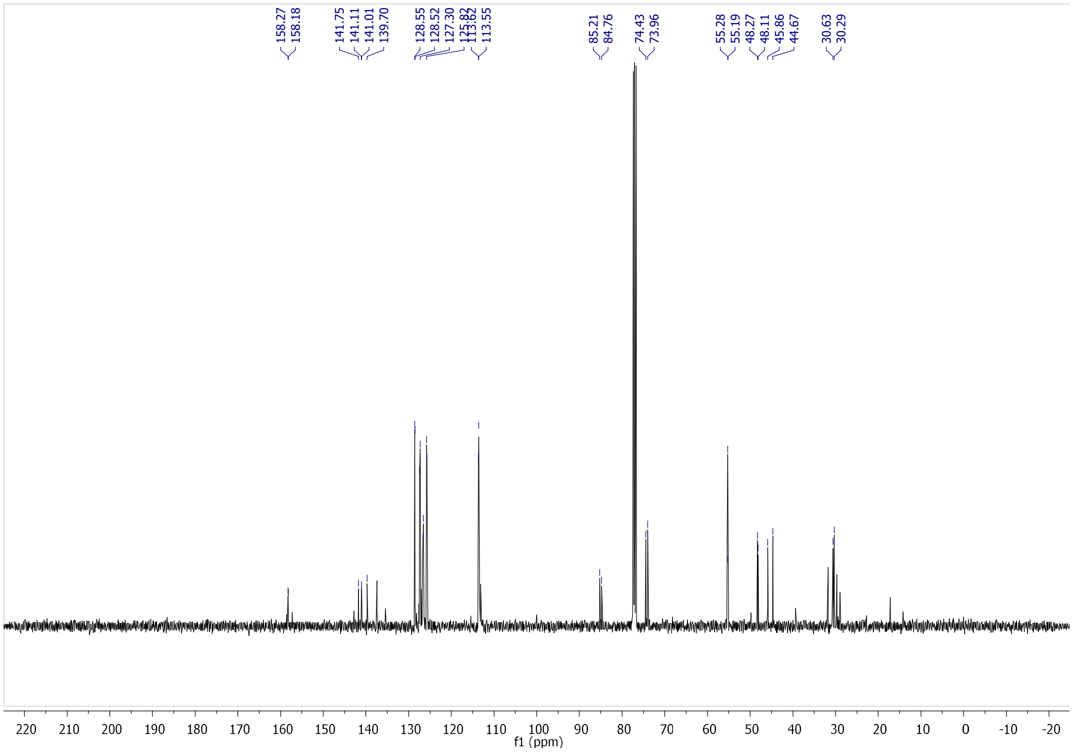

2,2,4-triphenyltetrahydrofuran (3ac)

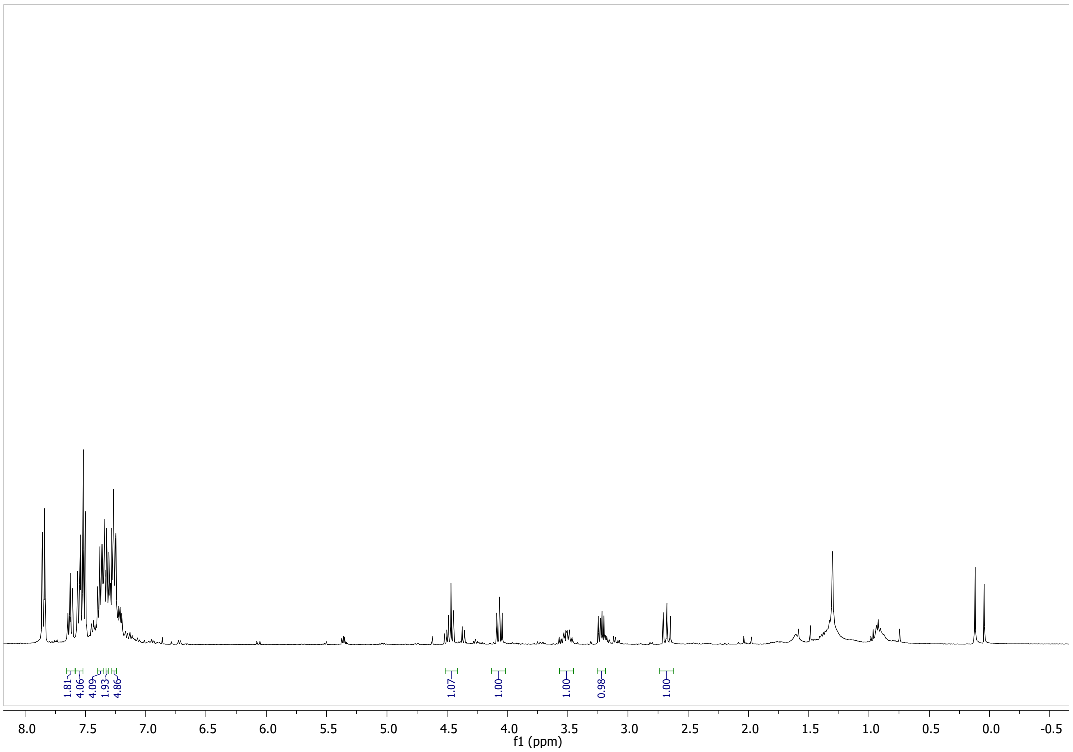

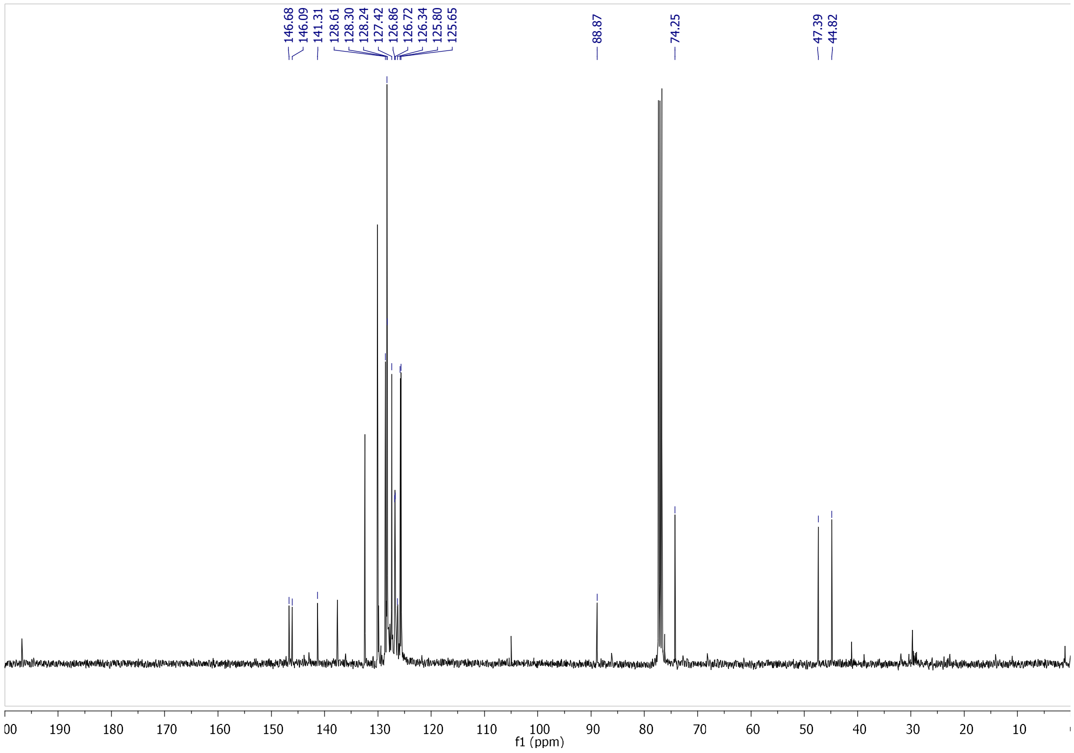

3-phenyl-1-oxaspiro[4.5]decane (3ag):

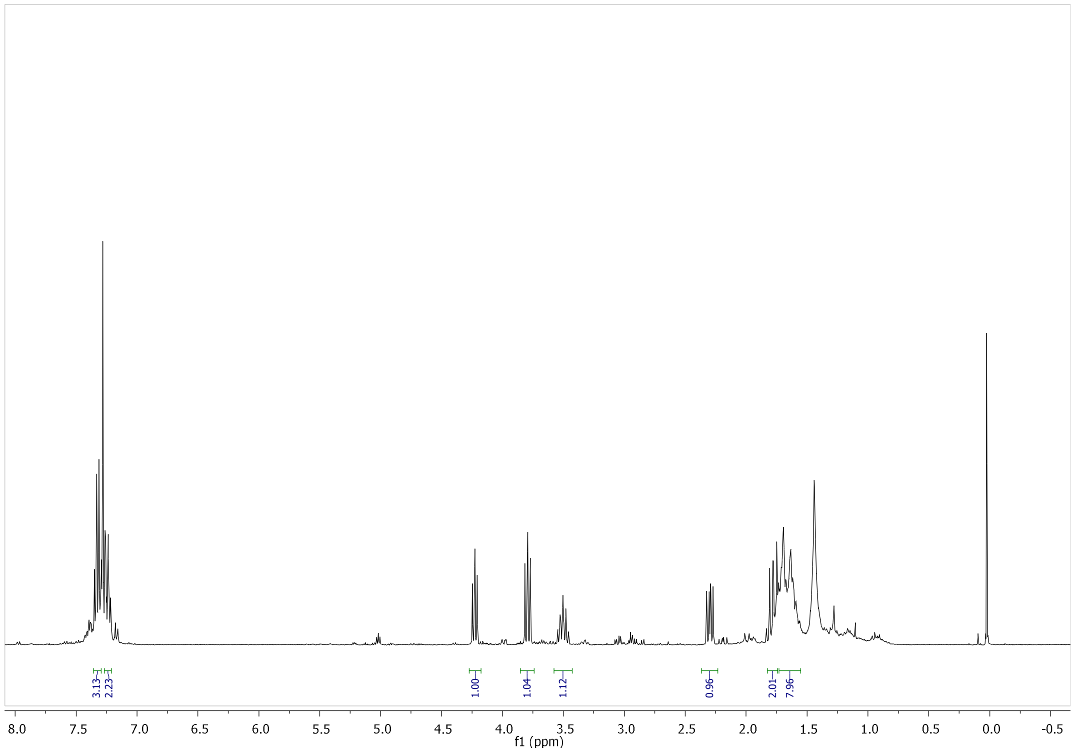

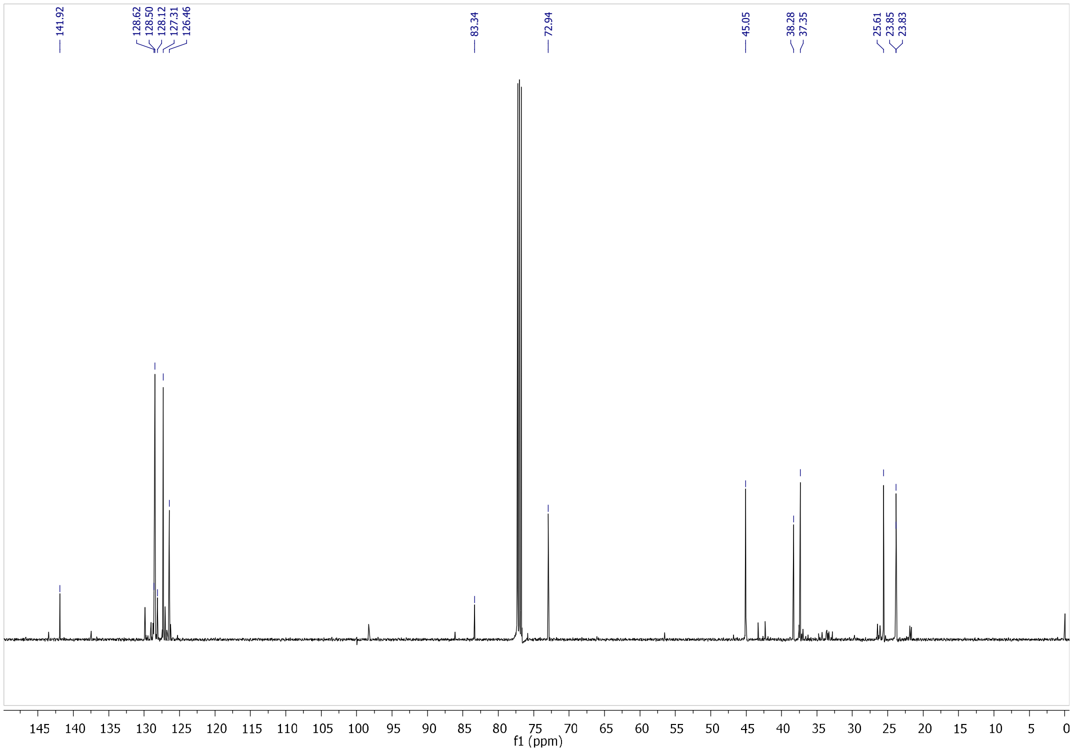

3,7a-diphenyloctahydrobenzofuran (3ai):

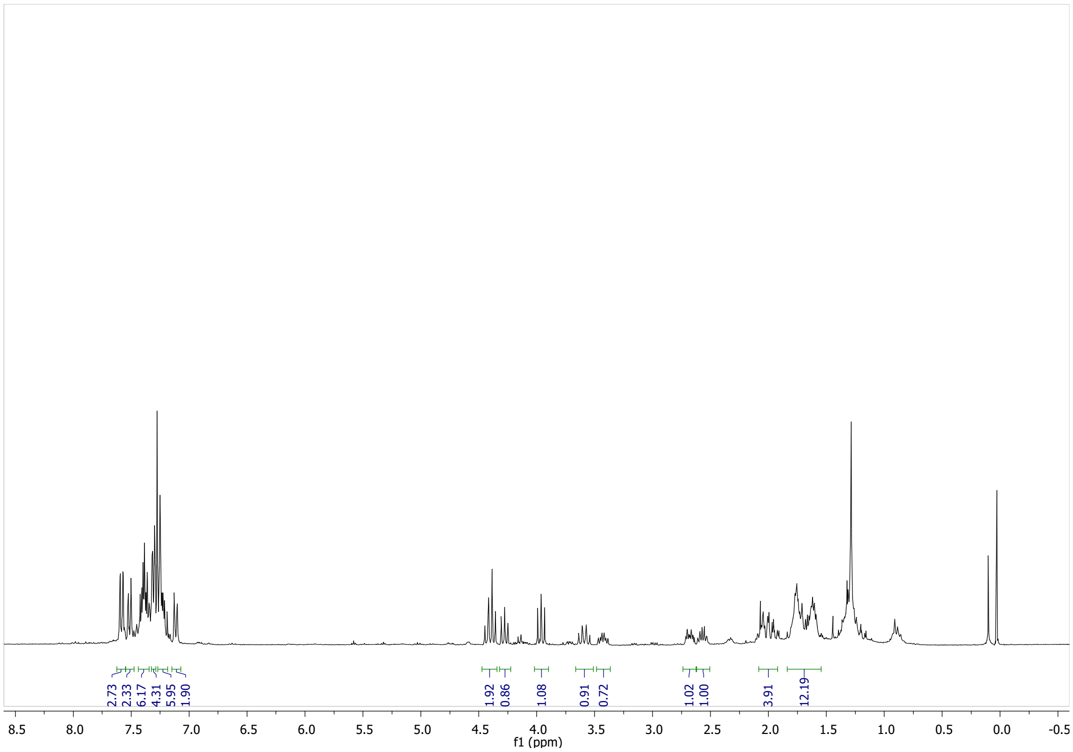

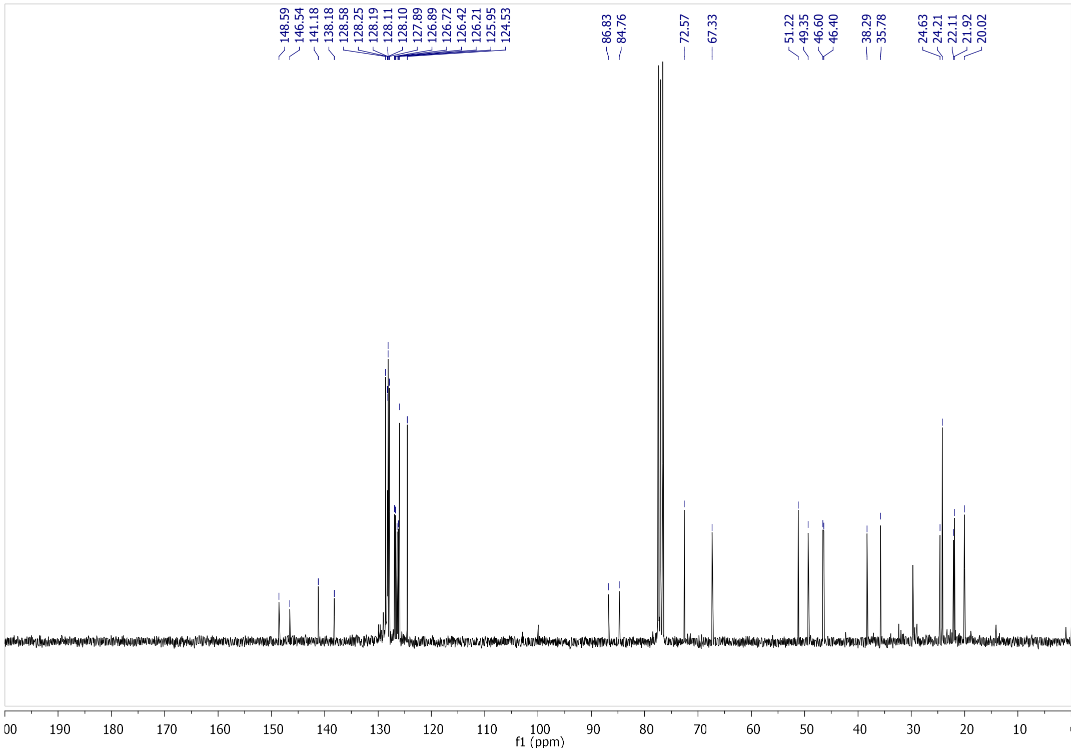

3-phenyl-2,3,3a,8b-tetrahydrofuro[3,2-b]benzofuran (3al):

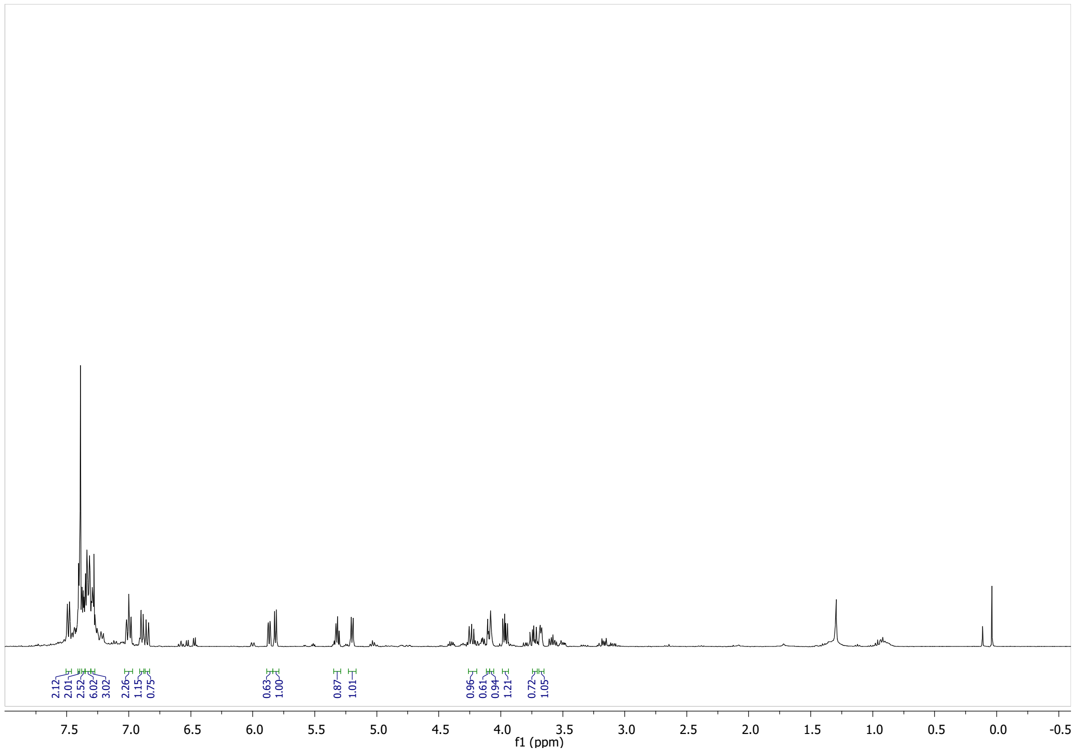

2-(4-methoxyphenyl)-2,4-dimethyl-4-phenyl tetrahydrofuran (3bb):

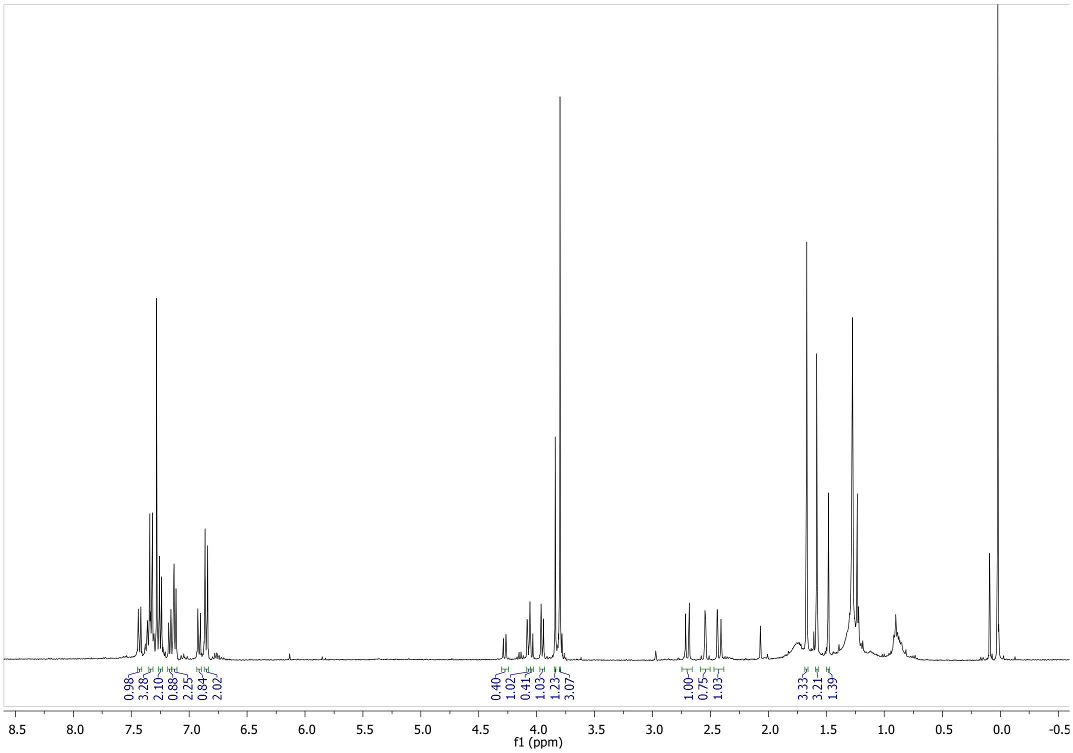

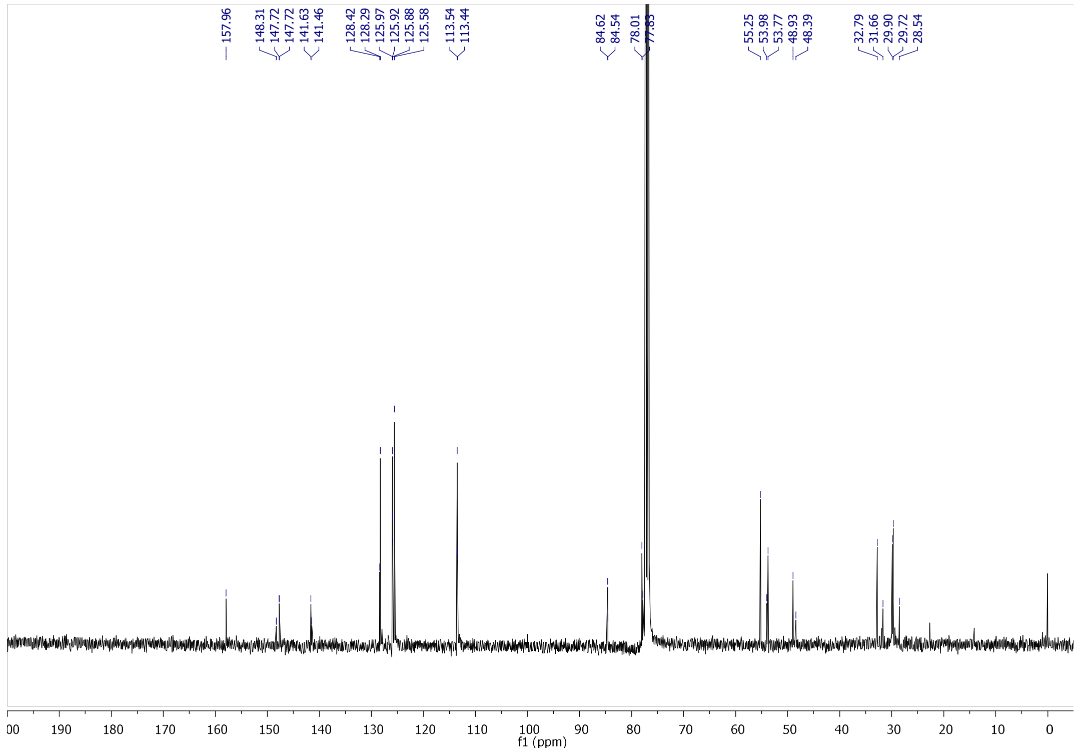

3-methyl-3-phenyl-1-oxaspiro[4.5]decane (3bg):

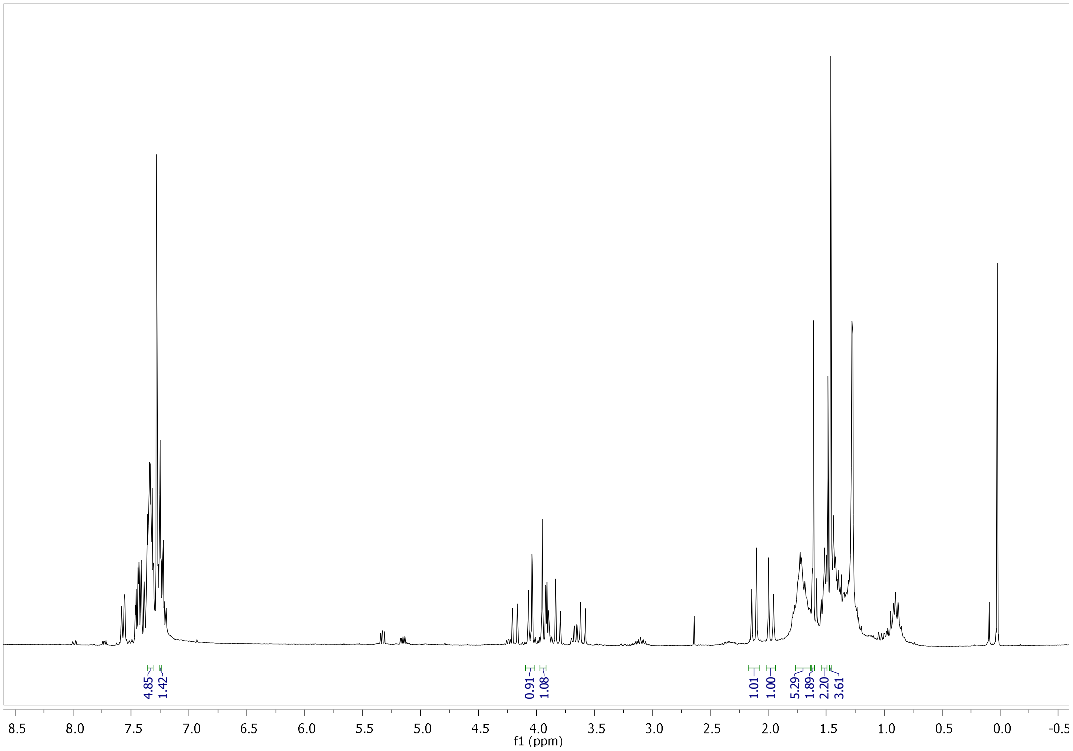

3-methyl-3-phenyl-2,3,3a,8b-tetrahydrofuro [3,2-b] benzofuran (3bl):

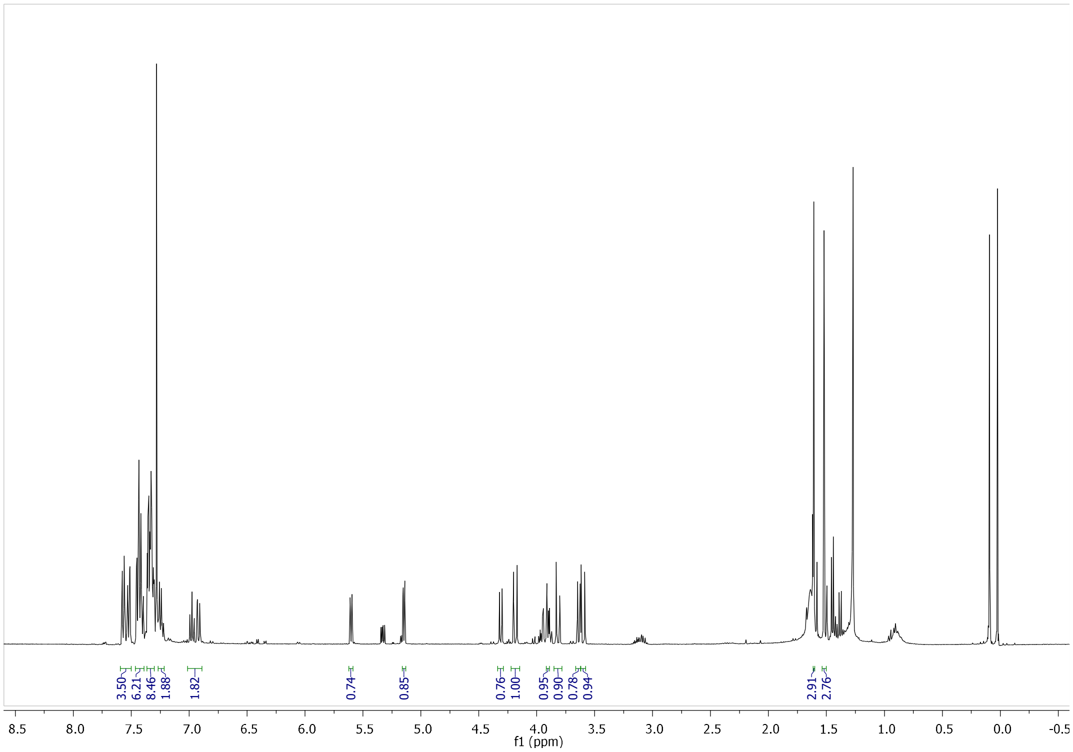

4a-phenyl-1,2,3,4,4a,4b,9b,10a-octahydrobenzofuro[3,2-b]benzofuran (3cl):

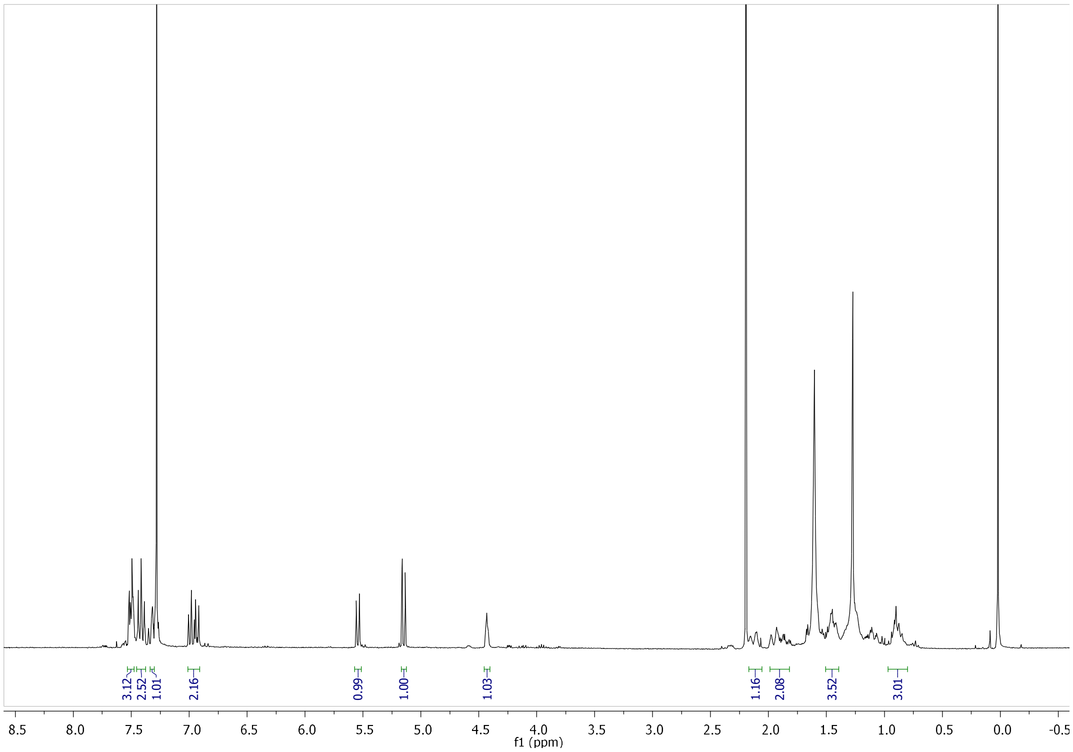

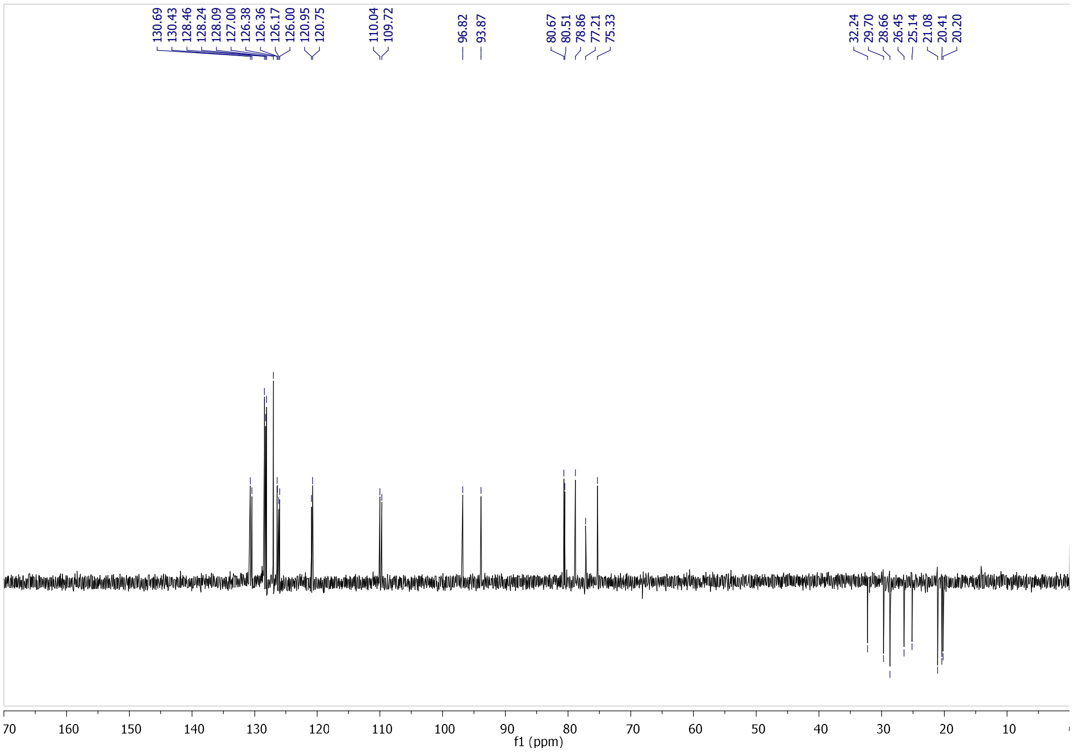

Ethyl - 3,5 - dimethyl - 3,5 - diphenyltetrahydrofuran - 2 - carboxylate (3da):

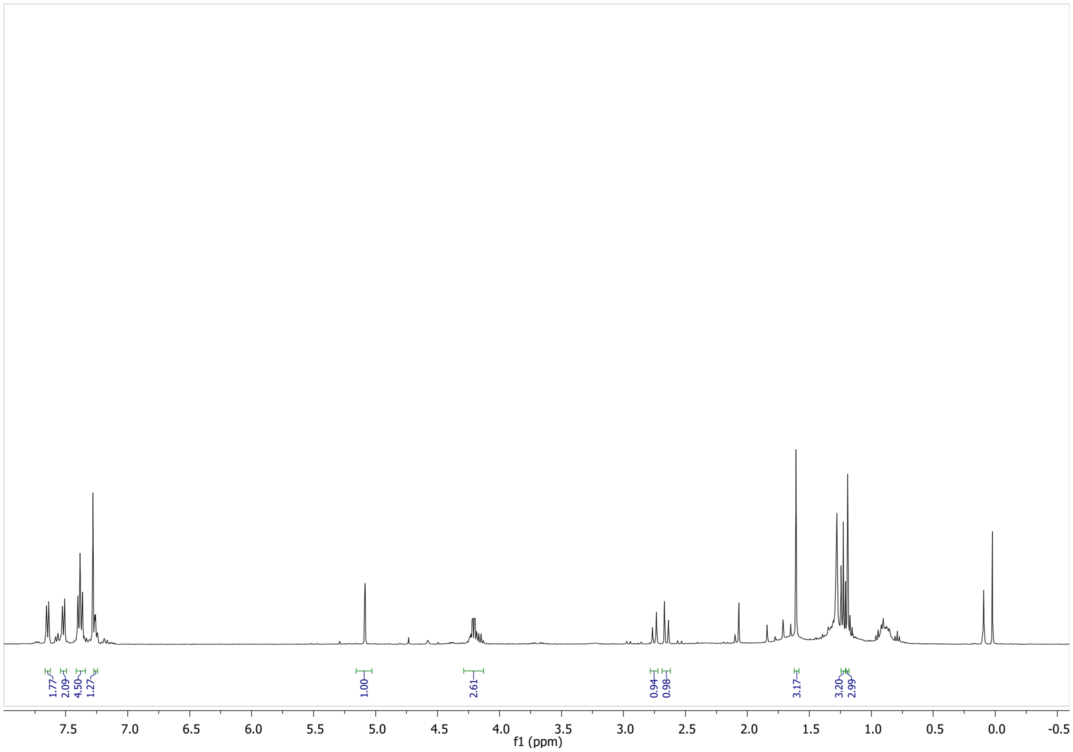

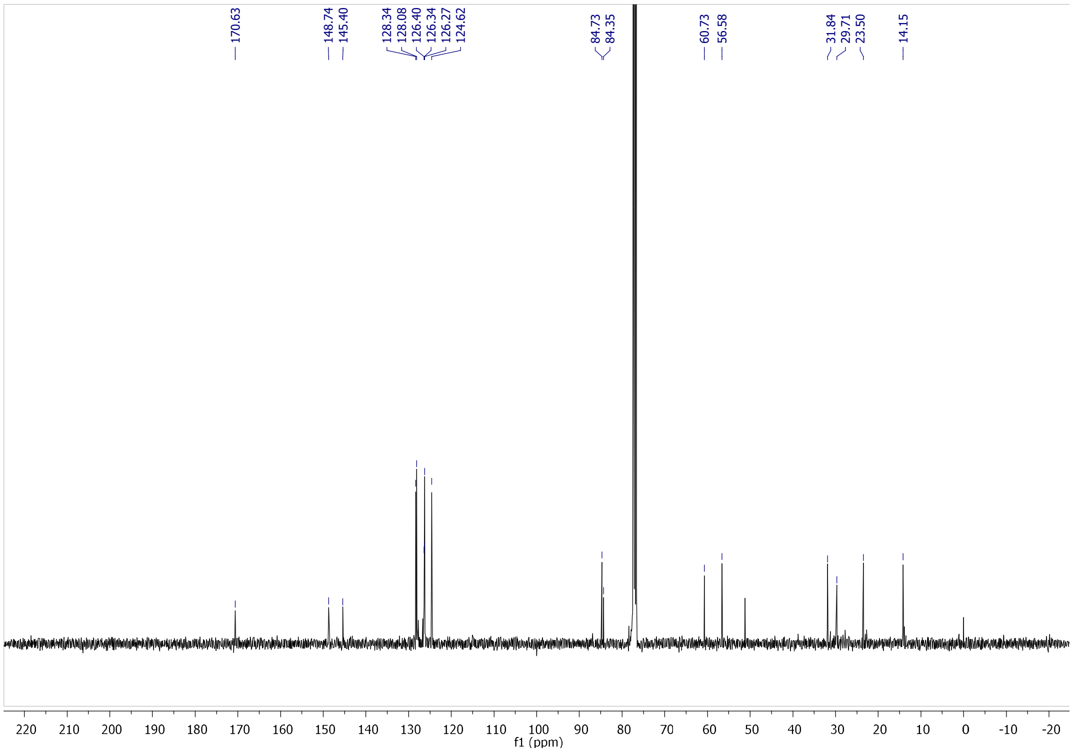

Ethyl 3-methyl-3,5,5-triphenyltetrahydrofuran-2-carboxylate (3dc):

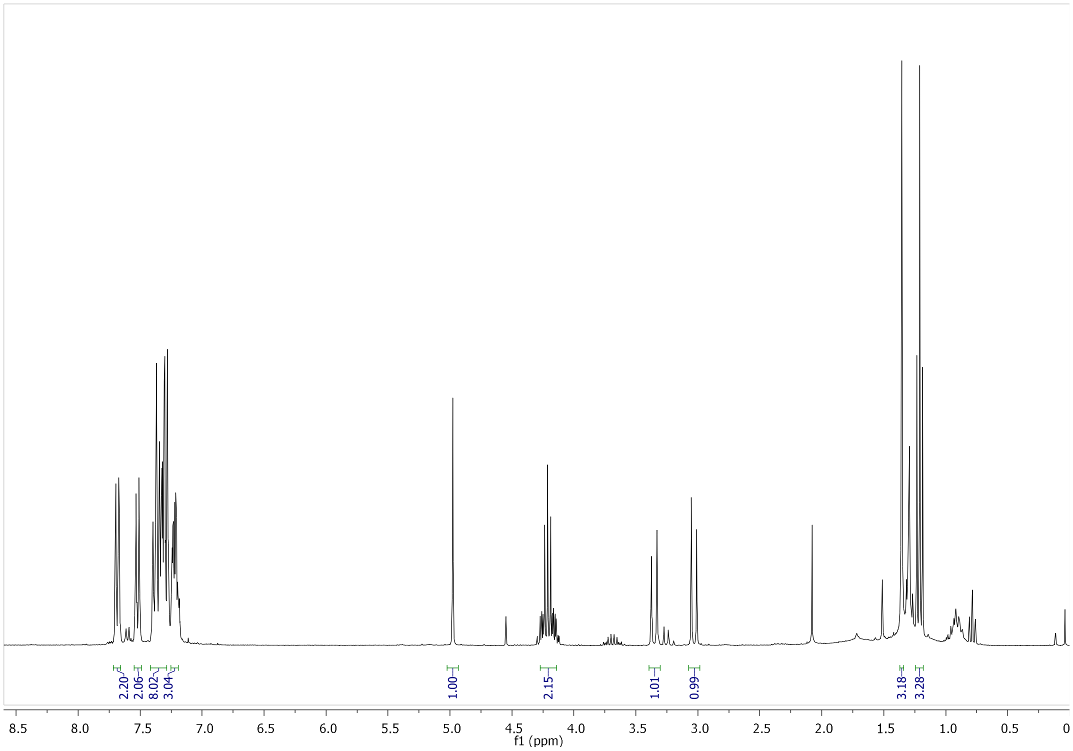

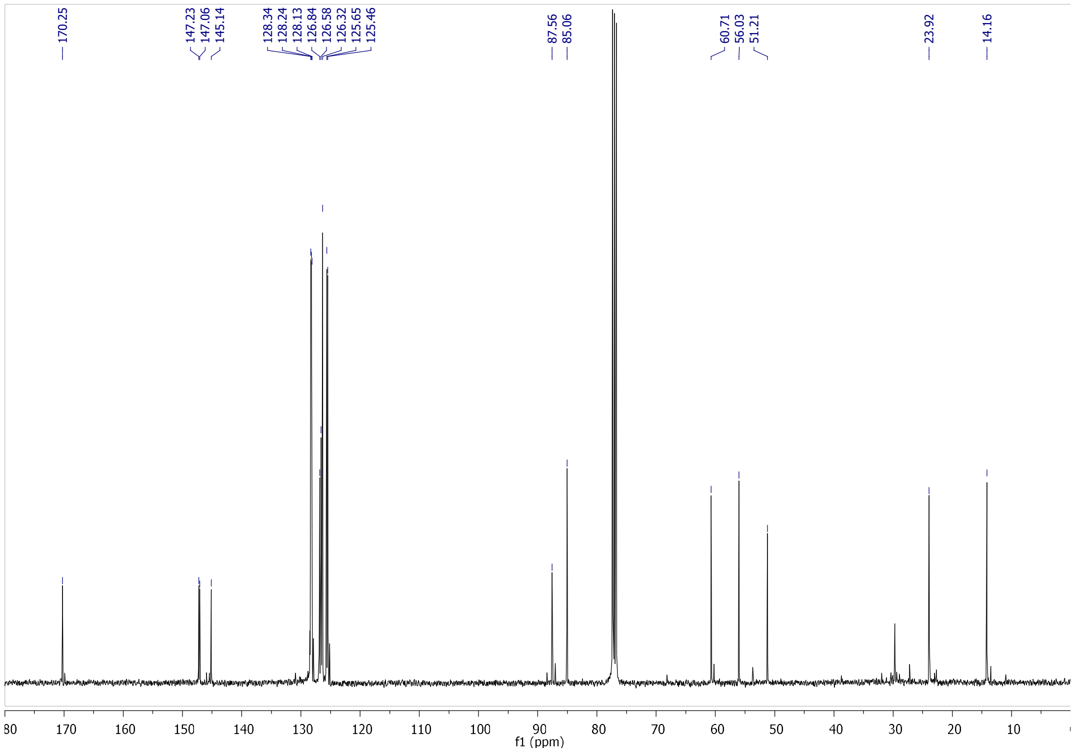

Ethyl-3-methyl-3,5-diphenyltetrahydrofuran-2-carboxylate (3dd):

Major isomer

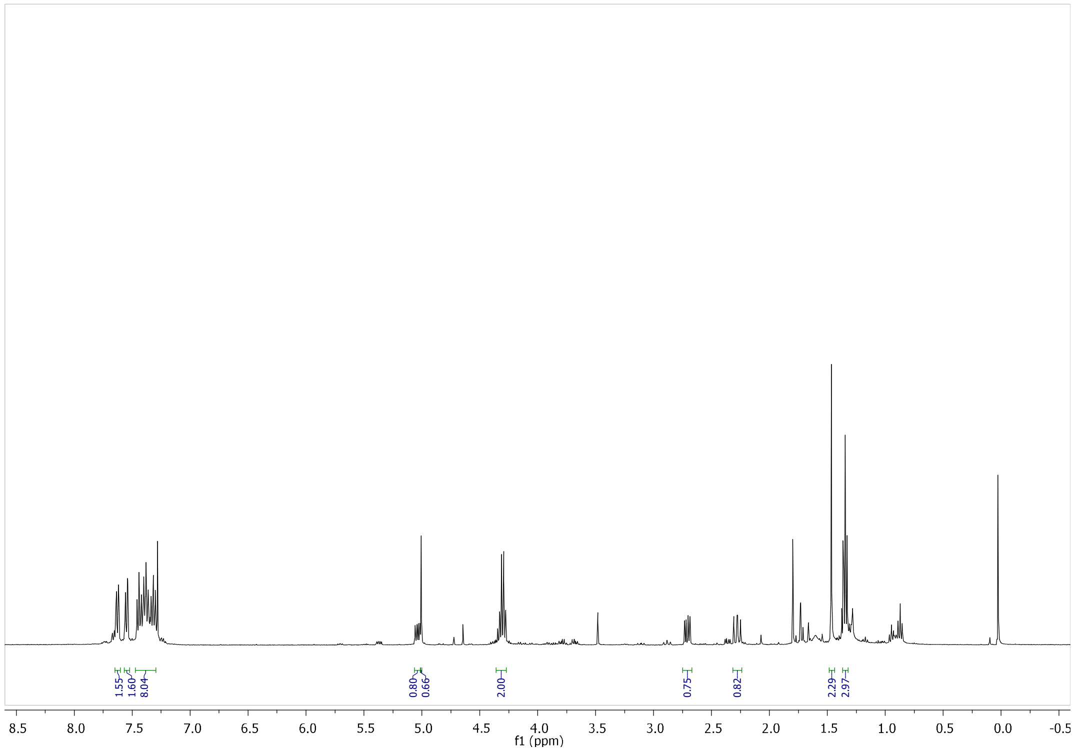

Minor isomer

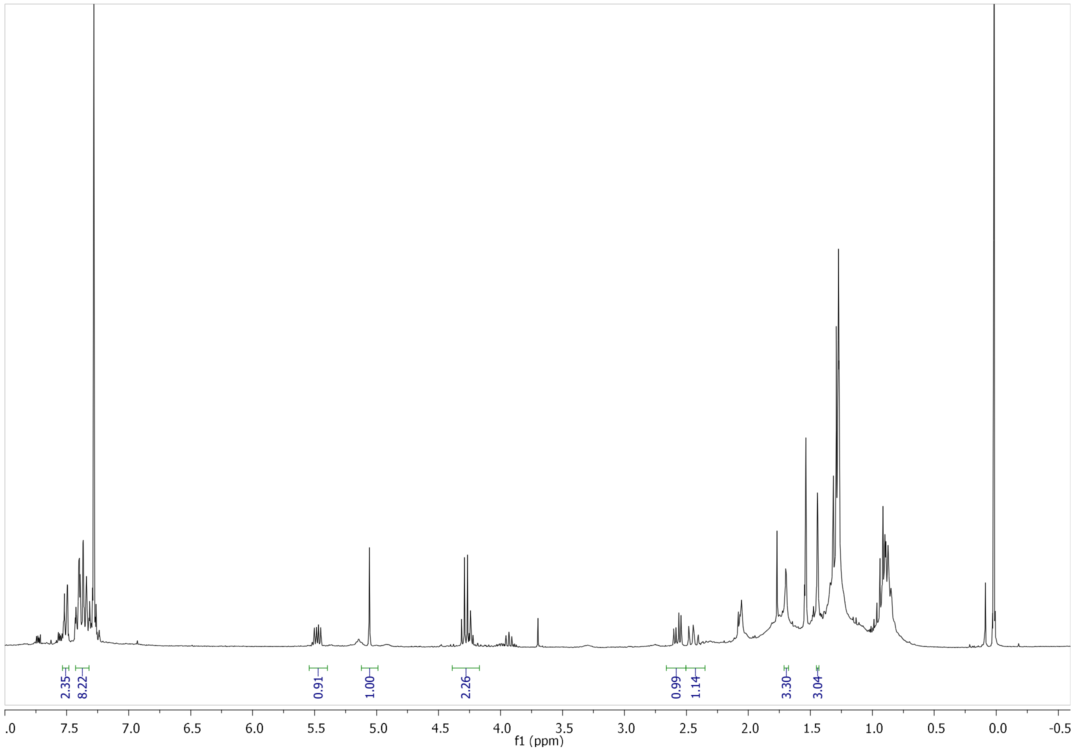

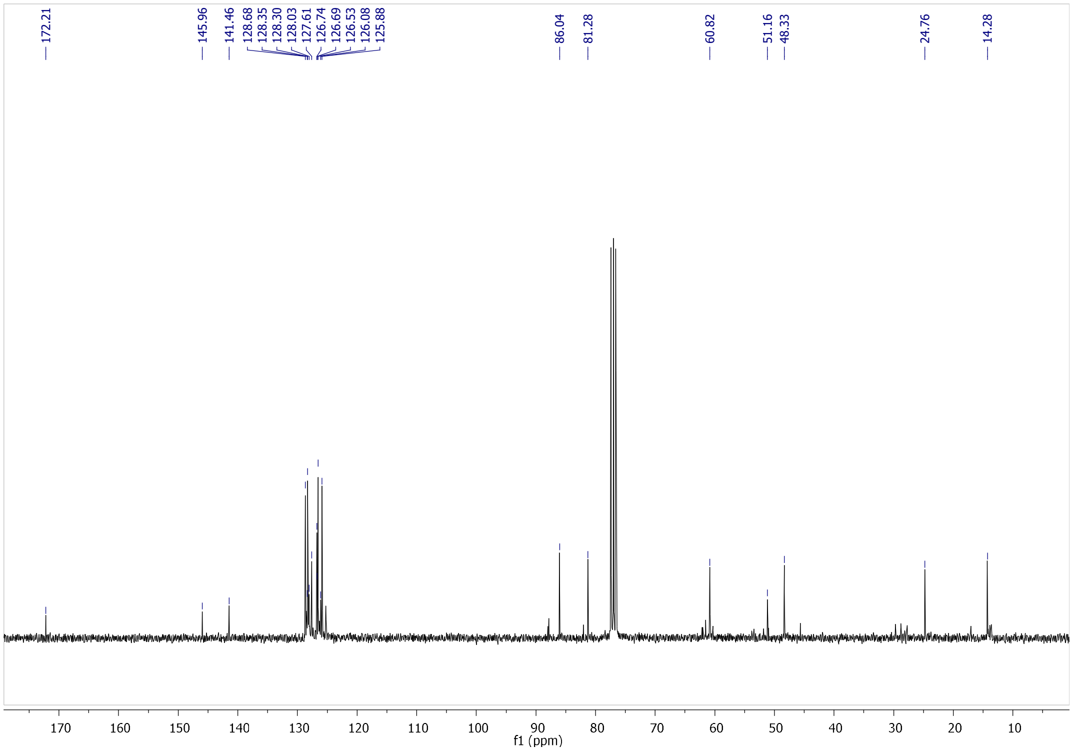

Ethyl 3-methyl-3-phenyl-2,3,3a,8b-tetrahydrofuro[3,2-b]benzofuran-2-carboxylate (3dl):

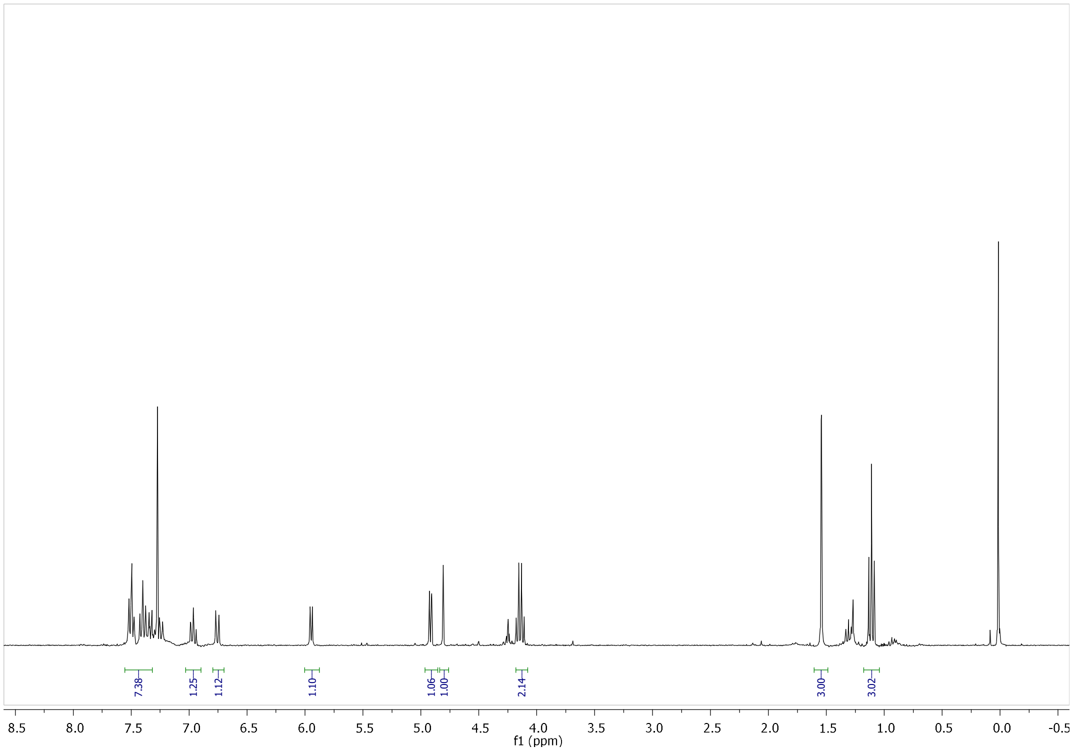

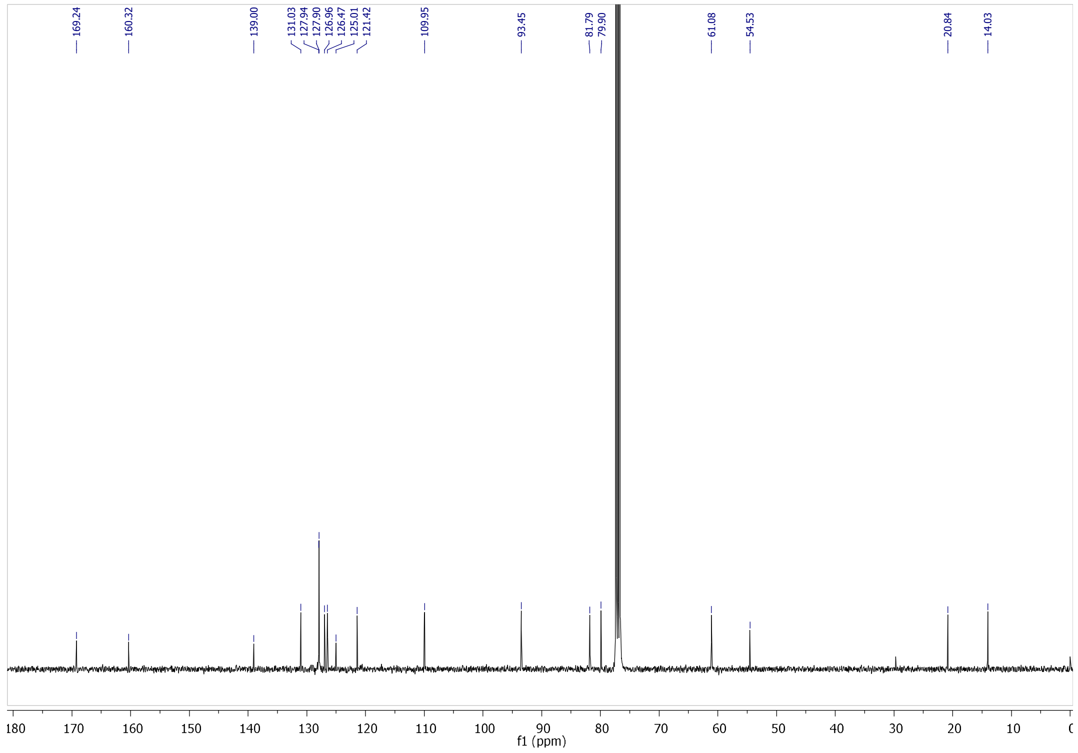

169.24
160.32
139.00
131.03
127.94
127.90
126.96
126.47
125.01
121.42
109.95
93.45
81.79
79.90
61.08
54.53
20.84
14.03
180 170 160 150 140 130 120 110 100 90 80 70 60 50 40 30 20 10 0
f1 (ppm)
